# GLP‐1 receptor agonists for treating obesity without diabetes: A systematic review and meta‐analysis of economic evaluations

**DOI:** 10.1111/dom.70322

**Published:** 2025-12-09

**Authors:** Teerapon Dhippayom, Manuel Meraz, Haeseon Lee, Chin Hur, John M. Inadomi, Sajesh K. Veettil, Jeffrey D. Dunn, Nathorn Chaiyakunapruk

**Affiliations:** ^1^ Department of Pharmacotherapy University of Utah College of Pharmacy Salt Lake City Utah USA; ^2^ The Research Unit of Evidence Synthesis (TRUES), Faculty of Pharmaceutical Sciences Naresuan University Phitsanulok Thailand; ^3^ Department of Medicine Columbia University, Irving Medical Center New York New York USA; ^4^ Department of Internal Medicine University of Utah, Spencer Fox Eccles School of Medicine Salt Lake City Utah USA; ^5^ School of Pharmacy IMU University Kuala Lumpur Malaysia; ^6^ School of Medicine Taylor's University Selangor Malaysia; ^7^ Department of Pharmacy Cooperative Benefits Group (CBG) Sandy Utah USA; ^8^ IDEAS Center, Veterans Affairs Salt Lake City Healthcare System Salt Lake City Utah USA

**Keywords:** cost‐effectiveness, glucagon‐like peptide‐1 receptor agonists, incremental net benefits, meta‐analysis, obesity

## Abstract

**Aim:**

To pool the incremental net benefits (INBs) of using glucagon‐like peptide‐1 receptor agonists (GLP‐1RAs) for treating obesity without diabetes.

**Materials and Methods:**

PubMed, Embase, EconLit, CEA Registry, ProQuest Dissertation and Theses Global were searched from inception to April 2024. Cost‐effectiveness studies were included if they reported economic outcomes of any GLP‐1RAs in the treatment of obesity without diabetes for a minimum time horizon of 5 years. Details of the study characteristics, economic model inputs, costs, and outcomes were extracted. Monetary units were converted to 2023 US dollars. INBs with 95% confidence interval (CI) were pooled using a random‐effects model. Statistical heterogeneity between studies was assessed using the *I* ^2^ statistic. The outcome was INB, calculated by multiplying the willingness‐to‐pay threshold by the difference in effectiveness between two interventions, then subtracting the difference in costs, with a positive INB indicating cost‐effectiveness.

**Results:**

Of 634 studies identified, 9 from high‐income countries (HICs) with 23 comparisons were included. The pooled INB demonstrated that semaglutide and liraglutide were not cost‐effective compared to no intervention (−$3659 [95% CI, −$74 379 to $67 062] and −$32 032 [95% CI, −$101 534 to $37 488], respectively) and lifestyle interventions (−$84 060 [95% CI, −$152 645 to −$15 475] and −$70 563 [95% CI, −$106 520 to −$34 605], respectively).

**Conclusions:**

GLP‐1RAs are generally not cost‐effective for obesity treatment in patients without diabetes in HICs from a healthcare/payer perspective. However, they may be cost‐effective in subgroups evaluated over longer time horizons. Most included studies focused on weight‐related outcomes, potentially underestimating the broader economic value of GLP‐1RAs. This pooled economic evidence may inform the decision‐making process.

## 1 INTRODUCTION

Obesity is a chronic medical condition that leads to numerous complications and independently increases the risk of cardiovascular disease and mortality.^1^ It was estimated that in 2022, approximately 1 billion people worldwide were living with obesity, which has more than doubled since 1990.^2^ The global burden of obesity and its associated comorbidities has elevated substantial healthcare expenditure, lessened individual quality of life, and caused loss of productivity.^3^ Effective treatments for obesity include lifestyle interventions, pharmacotherapy, and bariatric surgery.^4^

Glucagon‐like peptide 1 receptor agonists (GLP‐1RAs) are one effective treatment option recommended by various clinical practice guidelines for treating obesity.^5^, ^6^, ^7^ The current comparative evidence suggests that semaglutide provides substantially larger benefits in weight reduction than other drugs with a similar risk of adverse events.^8^ However, the costs of GLP‐1RAs are considered relatively high compared with other medications.^9^

Several economic evaluation (EE) studies showed inconsistent findings; some identified semaglutide as the most cost‐effective option,^10^, ^11^ while others reported high incremental cost‐effectiveness ratios (ICERs), suggesting it may not be cost‐effective.^10^, ^11^, ^12^ These mixed results make it unclear whether GLP‐1RAs are cost‐effective for obesity treatment. Crespo et al. proposed that a meta‐analysis of cost‐effectiveness studies could provide a quantitative summary of evidence to guide decision‐making.^13^ This approach has recently been endorsed by the WHO's Immunization and Vaccine‐related Implementation Research Advisory Committee (IVIR‐AC), especially for countries lacking context‐specific data,^14^ such as national estimates of direct and indirect healthcare costs or local willingness‐to‐pay thresholds, which are required to conduct tailored economic evaluations. In these settings, pooled cost‐effectiveness estimates from meta‐analyses may provide useful guidance for policy decision‐making. We conducted a systematic review and meta‐analysis of cost‐effectiveness studies and estimated the pooled incremental net benefits (INBs) to assess the cost‐effectiveness of using GLP‐1RAs for treating obesity in people without diabetes.

## 2 MATERIALS AND METHODS

This study followed the Preferred Reporting Items for Systematic Reviews and Meta‐Analysis Protocols (PRISMA).^15^ The study protocol was registered in PROSPERO (CRD42024555582).^16^

### 2.1 Search strategy

We searched the following bibliographic databases: PubMed, Embase, EconLit, Cost‐Effectiveness Analysis Registry by Tufts Medical Center, and ProQuest Dissertations and Theses Global. A comprehensive search was performed from database inception to 15 April 2024. The search strategies were constructed using the terms ‘GLP‐1 agonists’ and ‘cost‐effectiveness,’ which were applied as free‐text terms in the title/abstract fields and as thesaurus terms (e.g., MeSH in PubMed, Emtree in Embase) in each database. Full search strategies are presented in Appendix 1. References from studies that met the inclusion criteria in the database search were also reviewed to identify additional relevant studies.

### 2.2 Selection criteria

We included primary studies that met the following criteria: (1) conducted in patients without diabetes who had a body mass index (BMI) greater than or equal to 30 kg/m^2^; (2) compared GLP‐1RAs with no intervention, standard care, other pharmacologic or non‐pharmacologic interventions, or other GLP‐1RAs, and (3) reported cost per quality‐adjusted life years (QALYs) or life years (LY) for a minimum time horizon of 5 years to reflect the long‐term economic implications of GLP‐1RAs. The titles and abstracts of the search results were examined independently by two reviewers (Manuel Meraz and Haeseon Lee). Full‐text articles that passed the title/abstract screening process were subsequently assessed independently by two reviewers (Manuel Meraz and Haeseon Lee). Any disagreements and uncertainties about inclusion were addressed by a third reviewer (Teerapon Dhippayom).

### 2.3 Data extraction

A standardised data extraction sheet was developed based on the Consolidated Health Economic Evaluation Reporting Standard (CHEERS) checklist.^17^ Extracted data included characteristics of the population, interventions and comparators (including dose and administration), cost‐related details (currency and year), time horizon, study perspective, country income level (per the World Bank 2023 report), model used, discount rate, data source, incremental cost (ΔC), incremental outcomes (ΔE), incremental cost‐effectiveness ratio (ICER) and their dispersion (standard deviation [SD], standard error [SE], or 95% confidence interval [CI]), and willingness to pay (WTP) threshold. The cost‐effectiveness analysis plane with scatterplots representing ΔC and ΔE of the probabilistic sensitivity analysis was also retrieved and extracted using WebPlotDigitizer version 5 online application.^18^ Data were independently extracted by Manuel Meraz and Teerapon Dhippayom Any disagreement was solved by a discussion with Nathorn Chaiyakunapruk.

### 2.4 Risk of bias assessment

The risk of bias in the included studies was assessed using the modified Economic Evaluations Bias (ECOBIAS) checklist which consists of two main parts with 22 items.^19^ Each item was rated as yes, partly, no, unclear, or not applicable. Three important items that are relevant to the overall validity assessment and the study context included limited sensitivity analysis bias, wrong model bias, and bias related to treatment effects. Each study was categorised as low risk (answered ‘yes’ for all three items), high risk (answered ‘no’ for one or more items), or moderate risk (answered ‘partly’ or ‘unclear’ for one or more items). Manuel Meraz and Haeseon Lee independently assess risk of bias of the included studies, and any disagreements were resolved through discussion with Teerapon Dhippayom.

### 2.5 Outcome of interest and data preparation

The primary outcome of interest was INB of using GLP‐1RAs for obesity treatment. INB is a summary measure that combines both the cost and effectiveness of an intervention into a single value. It is calculated by multiplying the incremental effectiveness (measured in QALYs) by a predefined WTP threshold and then subtracting the incremental cost (Appendix 2). All INB values were calculated using a standardised WTP threshold of $100 000 per QALY, regardless of the threshold originally reported in each study. This was done to harmonise the interpretation of INB values and allow for valid pooling across studies. The $100 000 threshold was selected as it is a commonly accepted benchmark in high‐income countries, including the United States. A positive INB favours the intervention as cost‐effective, whereas a negative INB favours the comparator with the intervention being not cost‐effective.^20^ Data were prepared according to five scenarios (described in Appendix 2).^21^ All data reported in cost were converted to United States dollars (USD) in 2023 using the consumer price index (CPI)^22^ and purchasing power parity (PPP) conversion.^23^

### 2.6 Analysis

Meta‐analyses were performed to pool the INBs across studies stratified by country using the random‐effects model (DerSimonian and Laird method).^24^ Small‐study effects were assessed using a funnel plot and Egger's test. We used the chi‐squared test and *I* ^2^ to assess statistical heterogeneity across studies and assessed the heterogeneity among included studies based on variations in potential factors affecting the findings, including population characteristics, time horizon, model used, discount rate, and data source.

A series of pre‐specified sensitivity analyses were performed with the following conditions: (1) imputed variance using absolute values borrowed from similar studies (defined as studies with comparable patient populations, interventions and comparators, perspectives, and time horizons); (2) excluded studies categorised under scenario 5, which are those that lacked reported variance (as described in the data preparation section and Appendix 2); (3) excluded studies with incomprehensive sensitivity analyses; and (4) excluded studies with a treatment duration less than 5 years. We reviewed the sensitivity analyses conducted in the included studies and compiled a list of parameters that were tested. Key parameters were then identified based on their potential to influence cost‐effectiveness outcomes. Studies that did not include all of these key parameters in their sensitivity analyses were classified as having incomprehensive sensitivity analyses. In addition to the pre‐specified sensitivity analyses, two post hoc analyses were conducted: one using the restricted maximum likelihood (REML) method instead of the DerSimonian and Laird method to estimate between‐study variance, and another using a WTP threshold of $150 000 per QALY for the US studies. We also performed two sets of subgroup analyses based on (a) time horizon and (b) subject age. All data were prepared using Microsoft Excel version 365 and analysed by STATA version 18.0 (TX: Stata Corp).

## 3 RESULTS

### 3.1 Study selection

**FIGURE 1:**
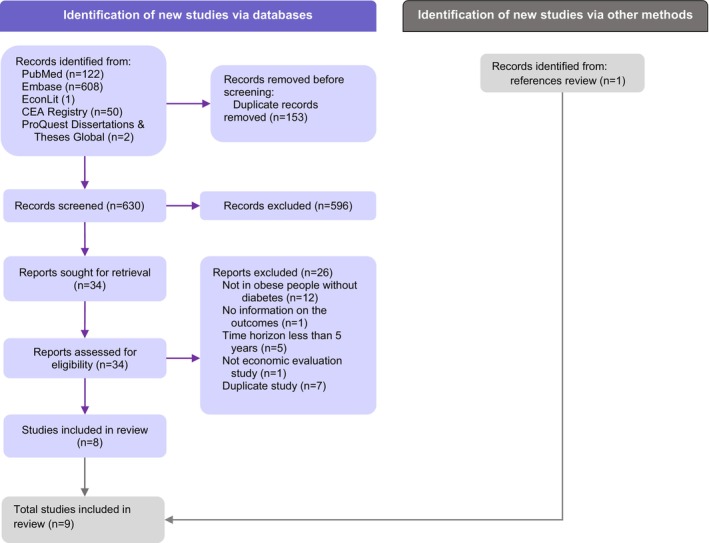
PRISMA flow diagram of selected articles.

We identified 634 articles from bibliographic database searches after duplicates were removed (Figure 1). A total of 30 articles were excluded after full text review (see the complete list with reasons for exclusion in Appendix 3). An additional study was identified by reviewing the references of 8 studies that met the inclusion criteria during the full‐text review, resulting in a total of 9 studies with 23 comparisons,^25^, ^26^, ^27^, ^28^, ^29^, ^30^, ^31^, ^32^, ^33^ all of which were model‐based economic analyses.

### 3.2 Study characteristics

All the studies were conducted in the United States, except for one, which was conducted in Switzerland.^32^ Seven studies explored the cost‐effectiveness of GLP‐1RAs in adult populations,^25^, ^26^, ^27^, ^28^, ^29^, ^32^, ^33^ whereas the other two focused on adolescents (Table 1).^30^, ^31^ Time horizon among the included studies ranged from 5 years^27^, ^29^, ^30^ to 42 years.^32^ Nearly all studies assumed the use of GLP‐1RAs throughout their entire study time horizon. However, the study by Kim et al. considered GLP‐1RAs use for only 2 years within their 30‐year time horizon.^28^ Among the 21 comparisons, six compared semaglutide with other treatments,^26^, ^28^, ^29^, ^30^, ^31^, ^33^ four compared liraglutide with other treatments,^26^, ^29^, ^30^, ^31^ and four compared semaglutide directly with liraglutide.^26^, ^28^, ^29^, ^31^ The included studies used various model‐based economic evaluations, such as the event‐driven decision analytic model,^32^ microsimulation model,^30^, ^31^, ^33^ and standard Markov model.^25^, ^26^, ^27^, ^28^, ^29^ The model of three studies covered diabetes‐related states as complications of obesity.^25^, ^26^, ^28^ A consistent discount rate of 3% was applied across all studies for both costs and outcomes.^25^, ^26^, ^27^, ^28^, ^29^, ^30^, ^31^, ^32^, ^33^ The INB and its variance for each study in every comparison are presented in Appendix 4.

**TABLE 1:** Characteristics of included studies.

Author	Country	Model type	Perspective	Patient age, years	Baseline BMI	Discount rate	Duration of using GLP‐1RA	Time horizon, years	Outcome measure	WTP threshold (USD)	Currency year	Intervention	Comparator	Funding
Atlas, 2022^25^	USA	Markov	Healthcare	45	38	Cost 3%, outcomes 3%	34	34	QALY	50 000, 100 000, 150 000	USD, 2022	SEMA SC 2.4 mg QW SEMA SC 2.4 mg QW SEMA SC 2.4 mg QW LIRA SC 3.0 mg OD	LI PHEN + TOPI NAL + BUP LI	Government grants and non‐profit foundations
Gomez‐Lumbreras, 2023^26^	USA	Markov	Payer	45	37.1 (F), 36.8 (M)	Cost 3%, outcomes 3%	40	40	QALY	150 000	USD, 2021	SEMA SC 2.4 mg QW SEMA SC 2.4 mg QW SEMA SC 2.4 mg QW LIRA SC 3.0 mg OD	PHEN + TOPI NAL + BUP LIRA NAL + BUP	No funding
Haseeb, 2024^27^	USA	Markov	Healthcare	45	37	Cost 3%, outcomes 3%	5	5	QALY	100 000	USD, 2022	SEMA SC 2.4 mg QW	ESG	NIH
Kim, 2022^28^	USA	Markov	Payer	46	37.9	Cost 3%, outcomes 3%	2	30	QALY	150 000	USD, 2021	SEMA 2.4 mg SEMA 2.4 mg SEMA 2.4 mg SEMA 2.4 mg SEMA 2.4 mg	LI No Tx PHEN + TOPI NAL + BUP LIRA 3.0 mg	Novo Nordisk Inc.
Lee, 2019^29^	USA	Markov	Healthcare	40	34.8	Cost 3%, outcomes 3%	5	5	QALY	100 000	USD, 2019	SEMA SC 0.4 mg OD SEMA SC 0.4 mg OD SEMA SC 0.4 mg OD SEMA SC 0.4 mg OD LIRA SC 3.0 mg OD LIRA SC 3.0 mg OD LIRA SC 3.0 mg OD	LI No Tx PHEN + TOPI LIRA LI No Tx PHEN + TOPI	Not report
Lim, 2023^30^	USA	Micro‐simulation	Healthcare	15	37	Cost 3%, outcomes 3%	5	5	QALY	100 000	USD, 2022	SEMA SC 2.4 mg QW LIRA SC 3.0 mg OD	LI LI	No specific grant
Mital, 2023^31^	USA	Micro‐simulation	Healthcare	15	36	Cost 3%, outcomes 3%	10	10	QALY	100 000	USD, 2023	SEMA SC 2.4 mg QW SEMA SC 2.4 mg QW LIRA SC 3.0 mg OD	No Tx LIRA No Tx	No funding
Nuijten, 2021^32^	Switzerland	Event‐driven decision	Payer	42	>30	Cost 3%, outcomes 3%	42	42	QALY	Not provided	CHF, 2019	LIRA SC 3.0 mg OD	No Tx	Nestlé Health Science
Saumoy, 2023^33^	USA	Semi‐ micro‐simulation	Healthcare	40	37	Cost 3%, outcomes 3%	30	30	QALY	100 000	USD, 2021	SEMA SC 2.4 mg QW	LI ESG	NIDDK

Abbreviations: BMI, body mass index; BUP, bupropion; ESG, endoscopic sleeve gastroplasty; F, female; LI, lifestyle intervention; LIRA, liraglutide; M, male; NAL, naltrexone; NIDDK, the National Institute of Diabetes and Digestive and Kidney Diseases; NIH, National Institutes of Health; OD, once daily; PHEN, phentermine; QALY, quality‐adjusted life year; QS, once weekly; SC, subcutaneous; SEMA, semaglutide; SG, sleeve gastrectomy; TOPI, topiramate; Tx, Treatment; USD, United States Dollar; WTP: willingness to pay.

Six^25^, ^27^, ^28^, ^30^, ^31^, ^33^ out of nine studies were justified as having conducted comprehensive sensitivity analyses, covering essential variables identified by our research team, which were treatment cost, efficacy, treatment discontinuation, weight regain following treatment discontinuation, utility, and complication costs (Appendix 5). Three studies,^26^, ^29^, ^32^ deemed not to have performed thorough sensitivity analyses, were excluded from the sensitivity analysis in our study.

### 3.3 Risk of bias of included studies

The overall risk of bias was low for half of the included studies (*n* = 5),^25^, ^27^, ^28^, ^30^, ^33^ moderate for three studies,^26^, ^29^, ^31^ and high for one study^32^ (Appendix 6). The main reason for the moderate and high risk of bias was due to limited sensitivity analysis.

### 3.4 Pooling of INB

#### 3.4.1 GLP‐1RAs versus lifestyle interventions

**FIGURE 2:**
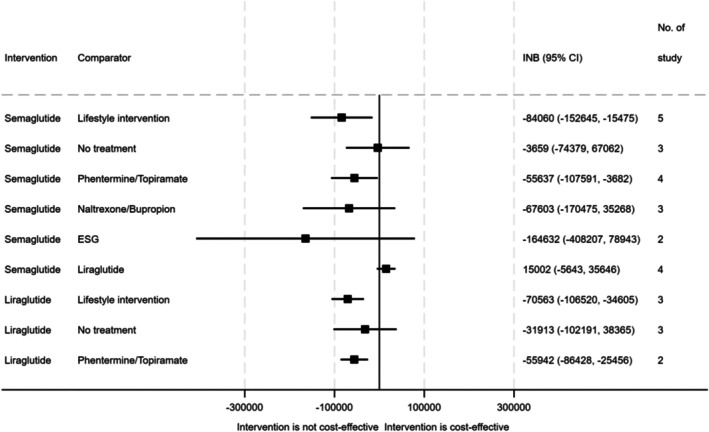
INB summary of GLP‐1RAs comparisons. CI, confidence interval; ESG, endoscopic sleeve gastroplasty; GLP‐1RAs, glucagon‐like peptide 1 receptor agonist; INB, incremental net benefit.

The pooled INB of semaglutide compared with lifestyle interventions indicated that it was statistically significantly not cost‐effective, with an INB of −$84 060 (95% CI −$152 645 to −$15 475), as shown in Figure 2 and Appendix 7. Liraglutide was also shown to be statistically significantly not cost‐effective when compared with lifestyle interventions, with a pooled INB of −$70 563 (95% CI −$106 520 to −$34 605). Findings from sensitivity analyses were consistent with the primary analyses, although some comparisons were not statistically significant (Appendix 8). Findings from subgroup analyses further indicated that GLP‐1RAs were not cost‐effective compared with lifestyle interventions across various groups with different study time horizons and age ranges (Appendix 9).

#### 3.4.2 GLP‐1RAs versus no treatment

When compared with no treatment, both semaglutide and liraglutide were not statistically significantly cost‐effective, with INBs of −$3659 (95% CI −$74 379 to $67 062) and −$31 913 (95% CI −$102 191 to $38 365), respectively. However, when limiting the analysis to US‐based studies by excluding the Swiss study, the results for liraglutide showed consistent and statistically significant findings, indicating it was not cost‐effective compared with no treatment, with an INB of −$53 230 (95% CI −$83 002 to −$23 458) (see Appendix 8). Nonetheless, findings from a sensitivity analysis that excluded studies with less comprehensive sensitivity favoured GLP1‐RAs, though this was not statistically significant, as indicated by a positive INB of $2339 (95% CI −$133 291 to $137 969),^28^, ^31^ as detailed in Appendix 8.

Additionally, findings from subgroup analyses showed variations across different groups. Specifically, in studies with a time horizon of more than 10 years, the pooled INB indicated that semaglutide was statistically significantly cost‐effective compared to no treatment ($63 453; 95% CI $108 843 to $116 023).^28^ In contrast, in studies with a time horizon of 10 years or less, semaglutide was not shown to be cost‐effective (−$26 520; 95% CI −$51 743 to −$1296).^29^, ^31^ Studies showed differing cost‐effectiveness outcomes between adult and adolescent populations, although these differences were not statistically significant. For adults, the INB was $16 730 (95% CI −$74 379 to $67 062),^28^, ^31^ while for adolescents, the INB was −$76 017 (95% CI −$185 567 to $33 533).^29^

#### 3.4.3 GLP‐1RAs versus other comparators

Both semaglutide and liraglutide were statistically significantly not cost‐effective compared to phentermine plus topiramate, with INBs of −$55 637 (95% CI −$107 591 to −$3682) and −$55 942 (95% CI −$86 428 to −$25 456), respectively (Figure 2). Additionally, semaglutide was not statistically significantly cost‐effective when compared with naltrexone plus bupropion (INB −$67 603; 95% CI −$170 475 to $35 268), and endoscopic sleeve gastrectomy (INB −$164 632; 95% CI −$408 207 to $78 943). Findings from the sensitivity and subgroup analyses were similar to the main analysis (Appendices 8 and 9).

#### 3.4.4 Semaglutide versus liraglutide

Semaglutide was found to be not statistically significantly cost‐effective compared to liraglutide, with a pooled INB of $15 002 (95% CI −$5643 to $35 646). Similar results were observed in the sensitivity analyses (Appendix 8). Subgroup analyses further indicated that semaglutide was not significantly cost‐effective compared to liraglutide across different study time horizons and age groups (Appendix 9).

To assess potential publication bias, we performed a visual inspection of funnel plots and applied Egger's test for each comparison. The results are presented in Appendix 10. None of the comparisons demonstrated evidence of asymmetry or statistically significant small‐study effects, suggesting no indication of publication bias in our meta‐analyses.

## 4 DISCUSSION

The growing burden of obesity underscores the need for accessible treatments. While GLP‐1RAs have demonstrated effectiveness for obesity treatment, their relatively high cost raises concerns about cost‐effectiveness, requiring evidence to support their use. Our study addressed this gap by pooling INBs from EEs of GLP‐1RAs, providing new insights into their cost‐effectiveness for treating obesity in people without diabetes. We found that both semaglutide and liraglutide are not cost‐effective compared to other interventions such as lifestyle modifications, phentermine‐topiramate, or naltrexone‐bupropion. These findings are consistent across studies and are due mainly to the high costs of these medications.

While the magnitude of pooled INBs varied, our findings showed a consistent trend: GLP‐1RAs were not cost‐effective compared with either no treatment or active anti‐obesity medications, including lifestyle interventions, phentermine‐topiramate, and naltrexone‐bupropion. This pattern was observed across the main analysis and sensitivity analyses. The only exception was in a subgroup comparison where GLP‐1RAs appeared cost‐effective relative to no treatment when modelled over extended time horizons (>10 years). These results highlight how cost‐effectiveness estimates can differ depending on the comparator and underscore the importance of distinguishing comparator type and time horizon when interpreting the economic value of GLP‐1RAs.

Our results differ from a previous systematic review of the economic evaluation of anti‐obesity drugs by Xue et al.,^34^ which found GLP‐1RAs to be cost‐effective for obesity management. This discrepancy may stem from differences in the included populations, as Xue et al. incorporated studies involving patients with T2D,^34^ where GLP‐1RAs address broader health complications and lead to greater cost offsets. In contrast, our study focused primarily on obesity treatment in people without diabetes. Moreover, the review by Xue et al. included only a small number of studies specifically evaluating GLP‐1RAs, most of which used short intervention durations of less than 5 years. These limited time horizons may underestimate long‐term costs and benefits. In addition, several studies adopted a societal perspective, incorporating indirect benefits such as productivity gains. These methodological differences likely contributed to the more favourable cost‐effectiveness outcomes reported by Xue et al. compared to our analysis including longer‐term, payer‐perspective studies. Including diabetes‐related health states in economic models likely amplifies the perceived benefits of obesity treatment, resulting in more favourable cost‐effectiveness outcomes in the original EE studies included in the previous review.

Several factors likely contributed to the findings that GLP‐1RAs are not cost‐effective for treating obesity in people without diabetes. One major factor is the high cost of these medications, particularly when compared to alternative weight management strategies such as lifestyle modifications, behavioural therapies, or other anti‐obesity medications. These alternatives typically have significantly lower costs and can result in meaningful, sustained weight loss over time.^35^ Additionally, most studies included in this review assumed continuous use of GLP‐1RAs throughout the study's time horizon, further compounding the overall cost. For obese people without diabetes, especially those at lower risk for obesity‐related complications, the benefits of weight loss may not justify the ongoing expense of long‐term treatment. Moreover, most simulation models may not account for broader benefits such as improved mental health, or productivity, which could lead to an underestimation of the overall value of GLP‐1RAs. Additionally, the indirect economic benefits of weight loss achieved through GLP‐1RAs, such as reductions in hospitalisations, surgeries, or medications for obesity‐related comorbidities, may take years to materialise. In the short term, these benefits are unlikely to offset the high costs of the medication, particularly for individuals who are not at high risk for obesity‐related health issues, or when such complications were not accounted for in the economic models.

The cost of GLP‐1RAs was consistently identified as a key driver of their cost‐effectiveness across included studies. Although most studies did not report whether prices reflected negotiated discounts or other pricing arrangements, and the cost inputs used were generally based on current or recent market prices. As such, the observed unfavourable cost‐effectiveness may partly reflect the high drug prices at the time of analysis. Notably, the majority of studies were conducted in the United States, where medication costs are typically higher than in many other countries We also acknowledge recent reductions in the market prices of GLP‐1RAs,^36^ may improve their cost‐effectiveness in future analyses. As pricing evolves, updated evaluations will be important to inform health policy and clinical decision‐making.

All included studies used a healthcare system or payer perspective, without considering a societal perspective that includes patient time costs or productivity losses. These broader costs can substantially affect cost‐effectiveness estimates, especially for obesity treatments that may reduce time lost from work or daily activities. Evidence from a previous review^37^ and CEA registries^38^ has shown that including such costs often results in more favourable cost‐effectiveness outcomes. Future evaluations should consider societal perspectives to better capture the full economic impact of GLP‐1RAs.

It is also worth exploring the potential cost‐effectiveness of GLP‐1RAs in specific subgroups of the obese population. For instance, individuals with severe obesity (BMI ≥40 or BMI ≥35 with comorbidities) or those with obesity‐related conditions such as hypertension or sleep apnoea may achieve more substantial health improvements from weight loss, potentially making these medications more cost‐effective for them. However, due to the limited number of studies and the lack of reported information, we were unable to perform subgroup analyses to examine this possibility in greater detail.

A specific model condition, as demonstrated in a study by Kim et al.,^28^ found semaglutide to be significantly cost‐effective compared to no treatment over a time horizon exceeding 10 years. This industry‐sponsored study applied several assumptions or methodologies that may have influenced the results. For example, it evaluated 2 years of semaglutide use over a 30‐year study time horizon that incorporated diabetes‐related health states. The 2‐year treatment duration was based on real‐world data showing most patients discontinue anti‐obesity medications (AOMs) within that period. The model assumed weight rebound after discontinuation until BMI returns to baseline, followed by natural weight gain. Although this reflects a plausible treatment pattern, the favourable cost‐effectiveness observed, particularly in the long‐term comparison of semaglutide versus no treatment, may be more strongly influenced by methodological factors such as time horizon, treatment duration, discontinuation patterns, and model structure, rather than reflecting true subgroup effects or biological differences. While Kim et al.'s study was assessed as low risk of bias for sponsorship using the ECOBIAS checklist, its unique methodological approach may have contributed to heterogeneity and influenced the pooled findings.

Furthermore, many included models assumed long‐term or lifelong adherence to GLP‐1RA therapy, which may not reflect real‐world usage patterns. In practice, discontinuation rates are high due to factors such as cost, side effects, and patient preferences.^39^, ^40^ These real‐world adherence issues may affect the actual effectiveness and economic value of GLP‐1RAs, particularly if early discontinuation limits the duration of health benefits.^41^ Incorporating these considerations is essential for improving the generalisability and accuracy of cost‐effectiveness estimates.

Our study identifies methodological variability as a significant challenge in economic evaluations of GLP‐1RAs. The included studies employed diverse modelling techniques, input parameters, and time horizons, contributing to high heterogeneity in some comparisons. Other potential sources of heterogeneity include variations in target populations (adults versus adolescents), assumptions regarding treatment duration, and the inclusion of diabetes‐related health states in some models but not others. Importantly, findings from sensitivity and subgroup analyses indicated that heterogeneity was reduced when studies with similar characteristics, such as comparable time horizons, age groups, or treatment duration, were analysed within the same subgroup. This suggests that heterogeneity in certain comparisons arose from structural and contextual variations rather than random error. Accordingly, while the pooled INB estimates provide useful summary measures of economic value, their reliability and generalisability depend on the degree to which decision contexts align with the modelling assumptions and structural characteristics of the included studies.

This variability reflects the broader challenge in generalising findings from individual studies to inform health policy and reimbursement decisions. For example, the study by Atlas et al.^25^ reported a wide 95% confidence interval for the INB in certain comparisons, likely due to differences in model assumptions such as long‐term treatment effects, weight regain, and input parameter values. Despite this, our sensitivity analyses excluding studies lacking comprehensive parameter exploration confirmed the robustness of the pooled findings. By applying a meta‐analytic approach using INB, we synthesised results across studies and provided a more generalisable estimate of cost‐effectiveness that accommodates variation in modelling frameworks and input assumptions. These results highlight the importance of adopting standardised methodologies to improve comparability across studies. Key recommendations include consistent definitions of health states, utility measures, and cost inputs, along with robust sensitivity analyses.

Our findings suggest that while GLP‐1RAs may not be the most cost‐effective option for the general population without diabetes, they could still be valuable in specific clinical scenarios. For example, semaglutide may be a reasonable choice for patients who require long‐term treatment or for whom alternative weight management options are unsuitable or unavailable. Moreover, the American Gastroenterological Association (AGA) guidelines advocate for personalised treatment plans considering comorbidities, patient preferences, and costs.^5^ In this context, semaglutide and liraglutide could be considered for patients whose clinical profiles align with these criteria, despite their higher economic burden. However, discontinuation of GLP‐1RAs is a significant concern, driven by their high cost and side effects.^42^ These factors can reduce adherence, compromise effectiveness, and further increase the overall cost burden. Among the included studies, adherence assumptions were generally based on discontinuation rates from clinical trials.

The choice of a uniform WTP threshold of $100 000 per QALY was made to enhance comparability across the US studies. However, we acknowledge that this may not reflect thresholds used in all healthcare systems. We initially planned a subgroup analysis by income level to account for this variability, but all included studies were from high‐income countries, limiting this approach. To test the influence of threshold assumptions, we performed a sensitivity analysis using a higher WTP of $150 000 per QALY, which showed consistent results with the main analysis. Additionally, because GLP‐1RAs were generally not cost‐effective even at the $100 000 threshold, it is reasonable to infer that cost‐effectiveness would be even less favourable at the lower threshold, such as $50 000. This suggests that the findings are robust within the US contexts. However, cost‐effectiveness thresholds vary widely across countries, and may be much lower in resource‐limited settings. Therefore, interpreting these findings requires consideration of local healthcare priorities and WTP benchmarks.

Given that GLP‐1RAs were not found to be cost‐effective in high‐income countries, their economic value is likely to be less favourable in lower‐income settings, where healthcare budgets are more limited and WTP thresholds are considerably lower. In such settings, the high price of GLP‐1RAs could divert limited resources away from more cost‐effective public health interventions.

Despite methodological robustness, our study has some limitations. Most of the included studies focused exclusively on weight‐related outcomes when estimating effectiveness. While weight loss is an important endpoint in obesity treatment, GLP‐1RAs have also shown potential benefits in improving cardiovascular risk factors and metabolic parameters. The exclusion of these broader clinical outcomes may underestimate both the health benefits and the economic value of these medications. Future evaluations should incorporate a wider range of outcomes to provide a more comprehensive assessment of the value of GLP‐1RAs in obesity management.

In addition, none of the included studies adopted a societal perspective. This is an important limitation, as excluding indirect costs, such as productivity losses and patient time costs, may underestimate the broader economic value of GLP‐1RAs. For obesity treatments that may reduce work absenteeism and improve quality of life, inclusion of a societal perspective could lead to more favourable cost‐effectiveness estimates, as demonstrated in a study on diabetes where analyses from a societal perspective yielded more positive economic outcomes compared to those from a payer perspective.^43^ Future economic evaluations should incorporate this perspective to better reflect the full value of these therapies.

There was also a lack of reported relevant variance in some studies. To address this, we imputed or borrowed variance from similar studies under Scenario 5, as previously described,^21^ to retain as many relevant studies as possible in the pooled analysis. When studies with variance borrowed under Scenario 5 were excluded in sensitivity analyses, the findings were mixed. Some comparisons showed results consistent with the main analysis, while others exhibited a similar direction of effect but with greater statistical significance when Scenario 5 studies were excluded. This suggests that including studies without reported variance may have influenced the precision and statistical accuracy of the pooled estimates. Nonetheless, the overall direction of findings remained consistent across analyses, supporting the robustness of the main conclusions despite some variability in statistical significance.

Third, although there was considerable heterogeneity among the included studies, our aim was to estimate the pooled INB while acknowledging that true values may vary across settings. We addressed this variation through subgroup and sensitivity analyses and highlighted it in the discussion to support careful interpretation of the results. Lastly, another important methodological consideration in our analysis is the choice of a uniform WTP threshold. Although the original studies included in our review used varying thresholds (e.g., $50 000, $100 000, or $150 000 per QALY), we recalculated all INBs using a consistent threshold of $100 000 per QALY. This standardisation enhances comparability across studies and aligns with commonly cited decision thresholds in high‐income countries. Future studies may consider conducting threshold‐based subgroup analyses when more data are available.

In light of the observed variability and the nature of the economic outcomes analysed, an assessment of the certainty of evidence is important for contextualising our findings. Although there is no standard tool for evaluating the certainty of evidence in meta‐analyses of economic evaluations (MAEE), the GRADE framework, which was primarily developed for clinical outcomes, has limited applicability in this context. For example, the domain of imprecision requires a minimally important difference, which is not defined for economic outcomes such as INB. Based on key considerations, the certainty of our pooled evidence is unlikely to be high, mainly due to imprecision from the wide range of INBs. Indirectness is also present, as most studies were conducted in high‐income countries, particularly the United States. Although heterogeneity was observed, the direction of effect estimates was consistent, and variability was likely influenced by differences in model structures and assumptions rather than conflicting results.

## 5 CONCLUSION

Our study highlights that while GLP‐1RAs are effective for weight reduction, they are generally not cost‐effective for obesity treatment in people without diabetes compared to other interventions. This reflects the high cost of these medications, which limits their economic value despite their proven efficacy in weight reduction. However, GLP‐1RAs may be cost‐effective in certain subgroups, particularly when the benefits are evaluated over longer time horizons such as more than 10 years in comparison with no treatment. These findings offer potentially useful quantitative economic evidence to support decision making in obesity management.

## CONFLICT OF INTEREST STATEMENT

Nathorn Chaiyakunapruk received consulting honoraria from Novo Nordisk. And other authors declare no conflicts of interest.

## FUNDING INFORMATION

The research reported in this publication was supported (in part or in full) by the National Center for Advancing Translational Sciences of the National Institutes of Health under Award Number(s) UM1TR004409. The content is solely the responsibility of the authors and does not necessarily represent the official views of the National Institutes of Health.

## Supporting information


**Data S1.** Supporting Information.

## Data Availability

The data that support the findings of this study are available from the corresponding author upon reasonable request.
