# Supplementary material for: GLP‐1 receptor agonists for treating obesity without diabetes: A systematic review and meta‐analysis of economic evaluations
Source: Diabetes Obes Metab. 2025 Dec 9;28(2):1339–49. doi: 10.1111/dom.70322 (PMC12803668; doi:10.1111/dom.70322)
Supplement: Supplementary file 1 — Data S1. Supporting Information. [file DOM-28-1339-s001.docx]

**APPENDICES**

**Determining the incremental net monetary benefit of GLP-1 receptor agonists for obesity treatment in patients without diabetes: a systematic review and meta-analysis of economic evaluations**

**Table of Contents**

[Appendix 1: Search strategies (from inception to April, 2024) 1](#_Toc212133378)

[1.1 PubMed 1](#_Toc212133379)

[1.2 Embase 2](#_Toc212133380)

[1.3 EconLit 3](#_Toc212133381)

[1.4 Cost-effective Analysis Registry by Tufts Medical Center 4](#_Toc212133382)

[1.5 ProQuest Dissertations & Theses Global 5](#_Toc212133383)

[Appendix 2: Data preparation and currency conversion 6](#_Toc212133384)

[Appendix 3: List of excluded articles and reasons for exclusion 9](#_Toc212133385)

[3.1 Not in obese patients without diabetes (n=12) 9](#_Toc212133386)

[3.2 No information on the outcomes (n=1) 10](#_Toc212133387)

[3.3 Time horizon less than 5 years (n=5) 10](#_Toc212133388)

[3.4 Not economic evaluation studies (n=1) 10](#_Toc212133389)

[3.5 Duplicate studies (n=7) 10](#_Toc212133390)

[Appendix 4: Estimation of INB and its variance 12](#_Toc212133391)

[Appendix 5: Comprehensiveness of performing sensitivity analyses among included studies 14](#_Toc212133392)

[Appendix 6: Risk of bias of included studies 15](#_Toc212133393)

[Appendix 7: Pooled INB for each comparison 16](#_Toc212133394)

[7.1 Semaglutide vs lifestyle intervention 16](#_Toc212133395)

[7.2 Semaglutide vs no treatment 16](#_Toc212133396)

[7.3 Semaglutide vs phentermine/topiramate 16](#_Toc212133397)

[7.4 Semaglutide vs naltrexone/bupropion 17](#_Toc212133398)

[7.5 Semaglutide vs endoscopic sleeve gastroplasty 17](#_Toc212133399)

[7.6 Semaglutide vs liraglutide 17](#_Toc212133400)

[7.7 Liraglutide vs lifestyle intervention 18](#_Toc212133401)

[7.8 Liraglutide vs no treatment 18](#_Toc212133402)

[7.9 Liraglutide vs phentermine/topiramate 18](#_Toc212133403)

[Appendix 8: Sensitivity analyses 19](#_Toc212133404)

[Appendix 9: Subgroup analyses 20](#_Toc212133405)

[Appendix 10: Publication bias assessment for each comparison 21](#_Toc212133406)

[10.1 Semaglutide vs lifestyle intervention 21](#_Toc212133407)

[10.2 Semaglutide vs no treatment 21](#_Toc212133408)

[10.3 Semaglutide vs phentermine/topiramate 22](#_Toc212133409)

[10.4 Semaglutide vs naltrexone/bupropion 22](#_Toc212133410)

[10.5 Semaglutide vs endoscopic sleeve gastroplasty 23](#_Toc212133411)

[10.6 Semaglutide vs liraglutide 23](#_Toc212133412)

[10.7 Liraglutide vs lifestyle intervention 24](#_Toc212133413)

[10.8 Liraglutide vs no treatment 24](#_Toc212133414)

[10.9 Liraglutide vs phentermine/topiramate 25](#_Toc212133415)

# Appendix 1: Search strategies (from inception to April, 2024)

## 1.1 PubMed

| **Search number** | **Query** | **Results** |
| --- | --- | --- |
| ***Obesity domain*** | | |
| #1 | Obesity[MeSH] | 270,298 |
| #2 | “Weight Loss”[MeSH] | 51,062 |
| #3 | Overweight[MeSH] | 281,952 |
| #4 | Obes*[Title/Abstract] | 405,784 |
| #5 | “Body mass ind*”[Title/Abstract] | 252,515 |
| #6 | Overweight[Title/Abstract] OR “Over weight”[Title/Abstract] | 93,275 |
| #7 | Bodyweight[Title/Abstract] OR “Body weight”[Title/Abstract] | 258,326 |
| ***GLP-1 agonist domain*** | | |
| #8 | “Glucagon-Like Peptide 1”[MeSH] | 11,618 |
| #9 | Liraglutide[MeSH] | 2,636 |
| #10 | Exenatide[MeSH] | 2,947 |
| #11 | “GLP 1 agonist*”[Title/Abstract] OR “GLP-1 agonist*”[Title/Abstract] | 954 |
| #12 | “Glucagon like peptide 1 agonist*”[Title/Abstract] OR “Glucagon like peptide-1 agonist*”[Title/Abstract] | 381 |
| #13 | Exenatide[Title/Abstract] OR Bydureon[Title/Abstract] OR Byetta[Title/Abstract] | 2,444 |
| #14 | Liraglutide[Title/Abstract] OR Victoza[Title/Abstract] OR Saxenda[Title/Abstract] | 4,150 |
| #15 | Albiglutide[Title/Abstract] OR Eperzan[Title/Abstract] OR Tanzeum[Title/Abstract] | 249 |
| #16 | Lixisenatide[Title/Abstract] OR Lyxumia[Title/Abstract] OR Adlyxin[Title/Abstract] | 585 |
| #17 | Dulaglutide[Title/Abstract] OR Trulicity[Title/Abstract] | 777 |
| #18 | Semaglutide[Title/Abstract] OR Ozempic[Title/Abstract] OR Wegovy[Title/Abstract] OR Rybelsus[Title/Abstract] | 1,671 |
| ***Economic outcome domain*** | | |
| #19 | “Costs and Cost Analysis”[MeSH] | 269,799 |
| #20 | “Cost-Benefit Analysis”[MeSH] | 94,407 |
| #21 | Economics[MeSH] | 673,201 |
| #22 | “Quality-Adjusted Life Years”[MeSH] | 16,290 |
| #23 | “Cost analys*”[Title/Abstract] OR “Cost effica*”[Title/Abstract] OR “Cost effect*”[Title/Abstract] OR “Cost benefit*”[Title/Abstract] OR “cost utility”[Title/Abstract] | 210,129 |
| #24 | “Economic evaluation”[Title/Abstract] | 13,933 |
| #25 | “Quality adjusted life year*”[Title/Abstract] OR “Quality-adjusted life year*”[Title/Abstract] OR QALY[Title/Abstract] OR “Life year”[Title/Abstract] | 22,673 |
| #26 | “Incremental cost effectiveness ratio” [Title/Abstract] OR ICER[Title/Abstract] | 10,644 |
| #27 | “Incremental net benefit”[Title/Abstract] OR INB[Title/Abstract] | 411 |
| ***Combining all domains*** | | |
| #28 | OR/1-7 | 853,204 |
| #29 | OR/8-18 | 17,509 |
| #30 | OR/19-27 | 819,730 |
| #31 | AND/28-30 | 122 |

## 1.2 Embase

| **Search number** | **Query** | **Results** |
| --- | --- | --- |
| ***Obesity domain*** | | |
| #1 | 'obesity'/exp | 704,040 |
| #2 | 'body weight loss'/exp | 246,155 |
| #3 | obes*:ti,ab | 588,263 |
| #4 | 'body mass ind*':ti,ab | 363,060 |
| #5 | overweight:ti,ab OR ‘over weight’:ti,ab | 138,626 |
| #6 | bodyweight:ti,ab OR ‘body weight’:ti,ab | 349,100 |
| ***GLP-1 agonist domain*** | | |
| #7 | 'glucagon like peptide 1'/exp | 25,442 |
| #8 | 'liraglutide'/exp | 13,470 |
| #9 | 'semaglutide'/exp | 4,903 |
| #10 | ‘glp 1 agonist*’:ti,ab OR ‘glp-1 agonist*’:ti,ab | 1,785 |
| #11 | ‘glucagon like peptide 1 agonist*’:ti,ab OR ‘glucagon like peptide-1 agonist*’:ti,ab | 464 |
| #12 | exenatide:ti,ab OR bydureon:ti,ab OR byetta:ti,ab | 4,458 |
| #13 | liraglutide:ti,ab OR victoza:ti,ab OR saxenda:ti,ab | 7,635 |
| #14 | albiglutide:ti,ab OR eperzan:ti,ab OR tanzeum:ti,ab | 421 |
| #15 | lixisenatide:ti,ab OR lyxumia:ti,ab OR adlyxin:ti,ab | 949 |
| #16 | dulaglutide:ti,ab OR trulicity:ti,ab | 1,530 |
| #17 | semaglutide:ti,ab OR ozempic:ti,ab OR wegovy:ti,ab OR rybelsus:ti,ab | 2,905 |
| ***Economic outcome domain*** | | |
| #18 | 'cost'/exp | 420,988 |
| #19 | 'cost benefit analysis'/exp | 96,365 |
| #20 | 'economics'/exp | 252,097 |
| #21 | 'quality adjusted life year'/exp | 37,020 |
| #22 | 'cost analys*':ti,ab OR 'cost effica*':ti,ab OR 'cost effect*':ti,ab OR 'cost benefit*':ti,ab OR 'cost utility':ti,ab | 278,612 |
| #23 | 'economic evaluation':ti,ab | 17,071 |
| #24 | 'quality adjusted life year*':ti,ab OR 'quality-adjusted life year*':ti,ab OR qaly:ti,ab OR 'life year':ti,ab | 35,794 |
| #25 | 'incremental cost effectiveness ratio':ti,ab OR icer:ti,ab | 19,012 |
| #26 | 'incremental net benefit':ti,ab OR inb:ti,ab | 588 |
| ***Combining all domains*** | | |
| #27 | OR/1-6 | 1,456,774 |
| #28 | OR/7-17 | 42,357 |
| #29 | OR/18-26 | 894,519 |
| #30 | AND/27-29 | 608 |

## 1.3 EconLit

| **Search number** | **Query** | **Results** |
| --- | --- | --- |
| ***Obesity domain*** | | |
| #1 | TI obes* OR AB obes* | 2,176 |
| #2 | TI “body mass ind*” OR AB “body mass ind*” | 782 |
| #3 | TI (overweight OR "over weight") OR AB (overweight OR "over weight") | 971 |
| #4 | TI (bodyweight OR "body weight") OR AB (bodyweight OR "body weight") | 368 |
| ***GLP-1 agonist domain*** | | |
| #5 | TI ("glp 1 agonist*" OR "glp-1 agonist*") OR AB ("glp 1 agonist*" OR "glp-1 agonist*") | 2 |
| #6 | TI (exenatide OR bydureon OR byetta) OR AB (exenatide OR bydureon OR byetta) | 2 |
| #7 | TI (liraglutide OR victoza OR saxenda) OR AB (liraglutide OR victoza OR saxenda) | 2 |
| #8 | TI (albiglutide OR eperzan OR tanzeum) OR AB (albiglutide OR eperzan OR tanzeum) | 0 |
| #9 | TI (lixisenatide OR lyxumia OR adlyxin) OR AB (lixisenatide OR lyxumia OR adlyxin) | 0 |
| #10 | TI (dulaglutide OR trulicity) OR AB (dulaglutide OR trulicity) | 1 |
| #11 | TI (semaglutide OR ozempic OR wegovy OR rybelsus) OR AB (semaglutide OR ozempic OR wegovy OR rybelsus) | 3 |
| ***Combining all domains*** | | |
| #12 | OR/1-4 | 3,092 |
| #13 | OR/5-11 | 38 |
| #14 | AND/27-29 | 1 |

## 1.4 Cost-effective Analysis Registry by Tufts Medical Center

| **Search number** | **Query** | **Results** |
| --- | --- | --- |
| #1 | glp 1 AND obesity | 2 |
| #2 | exenatide AND obesity | 2 |
| #3 | liraglutide AND obesity | 6 |
| #4 | albiglutide AND obesity | 0 |
| #5 | lixisenatide AND obesity | 0 |
| #6 | dulaglutide AND obesity | 1 |
| #7 | semaglutide AND obesity | 3 |
| #8 | glp 1 AND “body mass index” | 10 |
| #9 | exenatide AND “body mass index” | 8 |
| #10 | liraglutide AND “body mass index” | 14 |
| #11 | albiglutide AND “body mass index” | 0 |
| #12 | lixisenatide AND “body mass index” | 2 |
| #13 | dulaglutide AND “body mass index” | 5 |
| #14 | semaglutide AND “body mass index” | 5 |
| #15 | glp 1 AND weight | 17 |
| #16 | exenatide AND weight | 10 |
| #17 | liraglutide AND weight | 21 |
| #18 | albiglutide AND weight | 0 |
| #19 | lixisenatide AND weight | 8 |
| #20 | dulaglutide AND weight | 11 |
| #21 | semaglutide AND weight | 11 |
| #22 | OR/1-21 | 50 |

## 1.5 ProQuest Dissertations & Theses Global

| **Search number** | **Query** | | **Results** | |
| --- | --- | --- | --- | --- |
| ***Obesity domain*** | | | | |
| #1 | title(obes*) OR abstract(obes*) | | 25,345 | |
| #2 | title("body mass ind*") OR abstract("body mass ind*") | | 8,569 | |
| #3 | title(overweight OR "over weight") OR abstract(overweight OR "over weight") | | 7,989 | |
| #4 | title(bodyweight OR "body weight") OR abstract(bodyweight OR "body weight") | | 13,539 | |
| ***GLP-1 agonist domain*** | | | | |
| #5 | title("glp agonist*" OR "glp-1 agonist*") OR abstract("glp agonist*" OR "glp-1 agonist*") | | 28 | |
| #6 | title("glucagon like peptide 1 agonist*" OR "glucagon like peptide-1 agonist*") OR abstract("glucagon like peptide 1 agonist*" OR "glucagon like peptide-1 agonist*") | | 1 | |
| #7 | title(exenatide OR bydureon OR byetta) OR abstract(exenatide OR bydureon OR byetta) | | 64 | |
| #8 | title(liraglutide OR victoza OR saxenda) OR abstract(liraglutide OR victoza OR saxenda) | | 61 | |
| #9 | title(albiglutide OR eperzan OR tanzeum) OR abstract(albiglutide OR eperzan OR tanzeum) | | 3 | |
| #10 | title(lixisenatide OR lyxumia OR adlyxin) OR abstract(lixisenatide OR lyxumia OR adlyxin) | | 5 | |
| #11 | title(semaglutide OR ozempic OR wegovy OR rybelsus) OR abstract(semaglutide OR ozempic OR wegovy OR rybelsus) | | 9 | |
| ***Economic outcome domain*** | | | |  |
| #12 | title("cost analys*" OR "cost effica*" OR "cost effect*" OR "cost benefit*" OR "cost utility") OR abstract("cost analys*" OR "cost effica*" OR "cost effect*" OR "cost benefit*" OR "cost utility") | 31,084 | |  |
| #13 | title("economic evaluation") OR abstract("economic evaluation") | 2,096 | |  |
| #14 | title("quality adjusted life year" OR "quality-adjusted life year" OR QALY OR "life year") OR abstract("quality adjusted life year" OR "quality-adjusted life year" OR QALY OR "life year") | 631 | |  |
| #15 | title("incremental cost effectiveness ratio" OR ICER) OR abstract("incremental cost effectiveness ratio" OR ICER) | 369 | |  |
| #16 | title("incremental net benefit" OR INB) OR abstract("incremental net benefit" OR INB) | 45 | |  |
| ***Combining all domains*** | | | | |
| #17 | OR/1-4 | 42,653 | |  |
| #18 | OR/5-11 | 139 | |  |
| #19 | OR/12-16 | | 33,832 | |
| #20 | AND/17-19 | | 2 | |

# Appendix 2: Data preparation and currency conversion

The following formulas are used for calculating INB and its variance (varINB).

INB = ΔE (K ― ICER) (1)

OR

INB = (K × ΔE) ― ΔC (2)

Var(INB) = K^2^σ^2^_ΔE_ + σ^2^_ICER_ (3)

OR

Var(INB) = K^2^σ^2^_ΔE_ + σ^2^_ΔC_ – 2Kρ_ΔCΔE_ (4)

Data required to generate INB and its variance as per equations 1 and 3 are ICER, variance of ICER, variance of incremental effectiveness (ΔE) and willingness-to-pay threshold (WTP). Data required to generate INB and its variance as per equations 2 and 4 are incremental cost (ΔC), and ΔE along with their variance, their covariance, and WTP threshold. In the model-based cost-effectiveness analysis, studies usually report point estimates of deterministic and/or probabilistic costs and outcomes. To generate above mentioned data, we used primarily the measures of central tendency and dispersion measures from PSA results for pooling, as it could better represent a real-life situation considering the distribution of all input variables. Recently, this method was further modified and devised a step-by-step data harmonization process to bring together inconsistently reported (e.g., money units) or missing data (e.g., data to estimate the variance of INB) from different EEs to facilitate performing meta-analyses as shown below:

Data was prepared according to 5 scenarios^[[1]](#footnote-1)^

***SCENARIO 1:*** The primary economic evaluation studies ideally report the point estimates and variances for every parameter required for the calculation of INB and its variance. Therefore, INB and its variance can be calculated directly from any of the formulas (1-4) provided above.

***SCENARIO 2:*** The study reports the means and measures of dispersion (95% CIs) of incremental costs & outcomes and ICER. The variance of the ICER can be calculated using the following formulas:


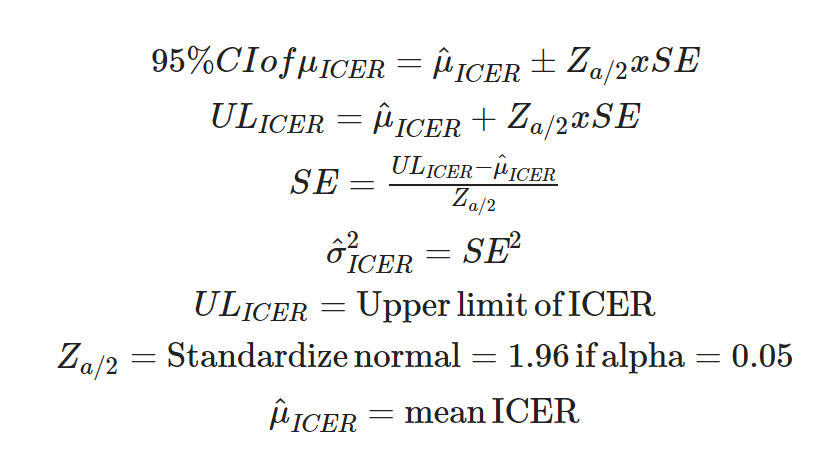


Once the variance of the ICER is calculated, the variance of the INB can be estimated using equation 3 provided above.

***SCENARIO 3:*** The study reports means, and 95% CI, SD/SE of costs/outcomes, or ΔC/ΔE, but does not provide the ICER or its variance. Data for Costs/ΔC and QALYs/ΔE are then used to simulate Costs/ΔC and QALYs/ΔE with 1000 replications using Monte Carlo methods with gamma and normal distributions for Costs/ΔC and QALYs/ΔE, respectively. We simulated costs and QALYs of intervention and comparator for 1000 replications separately; this then leads to calculating the ΔC, ΔE, and covariance between ΔC & ΔE. Data for ΔC and ΔE are assumed to be gamma and normal distributions. The covariance (σΔEΔC) between ΔC and ΔE as well as σ^2^ΔC & σ^2^ΔE can be then estimated. The INB and its variance can be further calculated using Eq. (1) and (4) provided above.

***SCENARIO 4:*** The study does not report any dispersion but does provide the CE plane graphs, a scatter plot of ΔC on the Y-axis and ΔE on the X-axis, in which individual values of ΔC and ΔE data can be manually extracted from the CE plane using Web-Plot-Digitizer software. Then, means of ΔC, ΔE, and their variances and covariances can be estimated accordingly. Finally, the INB and its variance can be estimated using Eq. (1) and (4).

***SCENARIO 5:*** The study reports neither any dispersion nor the CE-plane graph but only provides the deterministic analysis means (or point estimates) of costs, outcomes, and ICER. In such situations, the measures of dispersions can be borrowed from another similar study if they fulfill the following criteria:

- They are in the same stratum of country income,
- Similar model type and inputs (i.e., perspective, discounting, time horizon)
- They are similar in intervention, comparator, country region
- Their ICERs are not much different, e.g., ± 50% to 100%

If there is more than one study that meets the criteria, the average of the variances of those studies can be used.

There are two methods used in this study used when borrowing varINB data for scenario 5 studies. The relative data method was the preferred method used for the main analysis.

***Relative data method***: This method uses the above-mentioned criteria and then uses the formula listed below to make the varINB more like the values found in the scenario 5 study. Then the result is used as the varINB for the scenario 5 study.

$${(varINB}_{1}/{INB}_{1})\times{INB}_{2}={varINB}_{2}$$

***Absolute data method***: This method uses the above-mentioned criteria to use exact varINB data from another scenario 1-4 study for direct use in the scenario 5 study.

***Currency conversion:***

We need to standardize money units including WTP usually reported in different currencies (i.e., US $, CHF Fr.) and years by converting to purchasing power parity (PPP) adjusted to US$ for the latest year of analysis. For example, if a study reported cost, ICER, and thresholds in Swiss francs for 2018 and we plan to pool for the current year (e.g., 2023), this currency is firstly converted to 2023 Swiss francs using consumer price index (CPI) of that country (IMF database: <https://www.imf.org/en/Publications/WEO/weo-database/2023/October/download-entire-database>). Then, the Swiss franc 2023 value is to be converted to PPP adjusted US$ rate using conversion rates from the International Monetary Fund. In addition, GDP-based WTP threshold (K) values also need to be corrected for the current CPI 2023 year and PPP; however, standard/country-specific or fixed WTP values only need PPP correction.

Each currency will be converted to the United States Dollar (USD) in 2023 by using the consumer price index (CPI) and purchasing power parity (PPP).

$$Conversion factor \left( R \right)=(\frac{\mathrm{CPI}_{Fr.\left( in current year \right)}}{\mathrm{CPI}_{Fr.\left( in base year \right)}} \times\frac{1}{\mathrm{ppp}\left( in current year \right)} )$$

Costing data ($\Delta C, ICER, K^{*})$ were converted using the following equation:

$$Y_{\mathrm{ppp}\left( in current year \right)}=Y_{Fr.\left( in base year \right) \times}R$$

Variance of costing data ($\sigma_{\Delta C}^{2}, \sigma_{\mathrm{ICER}}^{2}$) were converted using the following equation:

$$V_{\mathrm{ppp}\left( in current year \right)}=V_{Fr.\left( in base year \right) \times}R^{2}$$

$Y_{ppp\left( in current year \right)}$ is the final monetary unit after adjusted to USD for the year 2023 using PPP conversion, $Y_{€\left( in base year \right)}$ is the monetary unit in study’s currency in base year of study, and R is the conversion factor for standardization of costing data.

# Appendix 3: List of excluded articles and reasons for exclusion

## 3.1 Not in obese patients without diabetes (n=12)

1. Armstrong S, Sen Gupta P, Amiel SA, Drummond RS, McGowan BM, Ryder REJ, et al. A UK cost-effectiveness analysis of the Endobarrier device in patients with Type 2 diabetes and obesity. Diabetic Medicine. 2018;35:169-70.
2. Barnett A, Subramanian G, Arnoldini S, Hunt B, Stentoft Hoxer C. Evaluation of the long-term cost-effectiveness of treatment switching from sitagliptin to liraglutide in subjects with Type 2 diabetes in the United Kingdom. Diabetic Medicine. 2018;35:147.
3. Dilla T, Alexiou D, Chatzitheofilou I, Ayyub R, Lowin J, Norrbacka K. The cost-effectiveness of dulaglutide versus liraglutide for the treatment of type 2 diabetes mellitus in Spain in patients with BMI ≥30 kg/m(2). J Med Econ. 2017;20(5):443-52.
4. Fonseca T, Clegg J, Caputo G, Norrbacka K, Dilla T, Alvarez M. The cost-effectiveness of exenatide once weekly compared with exenatide twice daily and insulin glargine for the treatment of patients with type two diabetes and body mass index ≥30 kg/m(2) in Spain. J Med Econ. 2013;16(7):926-38.
5. Goodall G, Costi M, Timlin L, Reviriego J, Sacristán JA, Smith-Palmer J, et al. [Cost-effectiveness of exenatide versus insulin glargine in Spanish patients with obesity and type 2 diabetes mellitus]. Endocrinol Nutr. 2011;58(7):331-40.
6. Gupta PS, Armstrong S, Amiel SA, Ryder RE, Pennington MW. Cost-effectiveness analysis of the endobarrier device in patients with type 2 diabetes. Diabetes. 2018;67:A335.
7. Malkin SJP, Russel-Szymczyk M, Psota M, Hlavinkova L, Hunt B. The Management of Type 2 Diabetes with Once-Weekly Semaglutide Versus Dulaglutide: A Long-Term Cost-Effectiveness Analysis in Slovakia. Adv Ther. 2019;36(8):2034-51.
8. Olivieri AV, Muratov S, Larsen S, Luckevich M, Chan K, Lamotte M, et al. Cost-effectiveness of weight-management pharmacotherapies in Canada: a societal perspective. Int J Obes (Lond). 2024.
9. Samyshkin Y, Guillermin AL, Best JH, Brunell SC, Lloyd A. Long-term cost-utility analysis of exenatide once weekly versus insulin glargine for the treatment of type 2 diabetes patients in the US. J Med Econ. 2012;15 Suppl 2:6-13.
10. Sandhu H, Xu W, Olivieri AV, Lübker C, Smith I, Antavalis V. Once-Weekly Subcutaneous Semaglutide 2.4 mg Injection is Cost-Effective for Weight Management in the United Kingdom. Adv Ther. 2023;40(3):1282-91.
11. Tan ECH, Yang MC. Cost-Effectiveness Analysis of Oral Semaglutide as the Second-Line and Third-Line Treatment for Type 2 Diabetes Patient. Value in Health. 2023;26(12):S52-S3.
12. Watkins JB, Minshall ME, Sullivan SD. Application of economic analyses in U.S. managed care formulary decisions: a private payer's experience. J Manag Care Pharm. 2006;12(9):726-35.

## 3.2 No information on the outcomes (n=1)

1. Valdez-Huerta R, Moreno D, Paladio Hernández JÁ. Cost-Effectiveness Analysis of Liraglutide for the Treatment of Obesity in Mexico. Value in Health. 2022;25(7):S350.

## 3.3 Time horizon less than 5 years (n=5)

1. Hu Y, Zheng SL, Ye XL, Shi JN, Zheng XW, Pan HS, et al. Cost-effectiveness analysis of 4 GLP-1RAs in the treatment of obesity in a US setting. Ann Transl Med. 2022;10(3):152.
2. Nuijten M, Marczewska A, Araujo Torres K, Rasouli B, Perugini M. A health economic model to assess the cost-effectiveness of OPTIFAST for the treatment of obesity in the United States. J Med Econ. 2018;21(9):835-44.
3. Nuijten M, Marczewska AM, Araujo Torres K, Morton M, Perugini M. Cost effectiveness of optifast® LCD as compared with liraglutide 3 mg and “no intervention” in Switzerland. Value in Health. 2017;20(9):A554-A5.
4. Perugini M, Marczewska AM, Araujo Torres K, Rasouli B, Nuijten M. OPTIFAST® meal replacement program for the treatment of obesity: A cost-effectiveness assessment from the employer perspective in the USA. Value in Health. 2018;21:S253.
5. Ruseva A, Ó Hartaigh B, Fabricatore A, Divino V, Coyle K, Doshi R, et al. RWD78 Characterization of Patients Treated with Semaglutide 2.4MG for Chronic Weight Management: A Retrospective Cohort Study. Value in Health. 2023;26(6):S375.

## 3.4 Not economic evaluation studies (n=1)

1. Valencia WM, Florez HJ. A new angle for glp-1 receptor agonist: the medical economics argument. Editorial on: Huetson P, Palmer JL, Levorsen A, et al. Cost-effectiveness of the once-daily glp-1 receptor agonist lixisenatide compared to bolus insulin both in combination with basal insulin for the treatment of patients with type 2 diabetes in Norway. J Med Econ. 2015;18(12):1029-31.

## 3.5 Duplicate studies (n=7)

1. Gómez-Lumbreras A, Tan MS, Villa Zapata L, Ilham S, Earl JC, Malone DC. EE2 A Cost-Effectiveness Analysis Comparing Obesity Drug Treatments from a U.S. Payer Perspective. Value in Health. 2022;25(7):S335.
2. Haseeb M, Hayat U, Jirapinyo P, Thompson CC. COST-EFFECTIVENESS ANALYSIS OF LAPAROSCOPIC SLEEVE GASTRECTOMY COMPARED TO SEMAGLUTIDE FOR WEIGHT LOSS IN PATIENTS WITH OBESITY. Gastroenterology. 2023;164(6):S-1482.
3. Haseeb M, Waqar M, Jirapinyo P, Thompson C. COST-EFFECTIVENESS ANALYSIS OF ENDOSCOPIC SLEEVE GASTROPLASTY COMPARED TO SEMAGLUTIDE FOR WEIGHT LOSS IN PATIENTS WITH OBESITY. Gastrointestinal Endoscopy. 2023;97(6):AB1167.
4. Haseeb M, Waqar M, Jirapinyo P, Thompson CC. COST-EFFECTIVENESS ANALYSIS OF INTRAGASTRIC BALLOONS COMPARED TO SEMAGLUTIDE FOR WEIGHT LOSS IN PATIENTS WITH OBESITY. Gastroenterology. 2023;164(6):S-711-S-2.
5. Lee M, Zhan T, Choi J, Klebanoff M, Kim D, Dayyeh BKA, et al. THE COST-EFFECTIVENESS OF PHARMACOTHERAPY IN OBESE PATIENTS. Gastroenterology. 2018;154(6):S-426.
6. Olivieri AV, Larsen S, Luckevich M, Chan K, Lamotte M. EE464 The Cost-Effectiveness of Subcutaneous Semaglutide 2.4MG Injection in the Management of Obesity in Canada Using the Core Obesity Model. Value in Health. 2022;25(7):S426.
7. Shah K, Kim K, Lien H, Atlas SJ, Moradi A, Beaudoin F, et al. EE8 Cost-Effectiveness of Long-Term Medication Therapy for Obesity Management. Value in Health. 2023;26(6):S61.

# Appendix 4: Estimation of INB and its variance

| **Author-year** | **Country** | **Adjusted WTP threshold (2023USD)** | **Delta effectiveness (per person)** | **Adjusted delta cost (per person) (2023USD)** | **Covariance** | **Adjusted ICER (2023USD)** | **INB** | **varINB** | **Scenario** |
| --- | --- | --- | --- | --- | --- | --- | --- | --- | --- |
| **Liraglutide vs lifestyle intervention** | | | | | | | | | |
| Atlas 2022 | US | 100,000 | 0.4100 | 204,525 | 974.0489 | 498,841 | -163,525 | 1,747,367,814 | 4 |
| Lee 2019 | US | 100,000 | -0.0725 | 52,290 | -31.7785 | -720,943 | -59,543 | 242,099,894 | 3 |
| Lim 2023 | US | 100,000 | 0.0317 | 55,200 | 50.0202 | 1,743,456 | -52,034 | 55,371,261 | 4 |
| **Liraglutide vs no treatment** | | | | | | | | | |
| Lee 2019 | US | 100,000 | 0.0476 | 55,660 | 15.0840 | 1,169,134 | -50,899 | 241,844,203 | 3 |
| Mital 2023 | US | 100,000 | 0.0912 | 110,795 | -571.6216 | 1,214,942 | -101,676 | 5,025,426,310 | 3 |
| Nuijten 2021 | CHF | 100,000 | 0.3990 | -5,746 | NA* | -14,401 | 45,646 | 2,256,102,001 | 5 |
| **Liraglutide vs phentermine plus topiramate** | | | | | | | | | |
| Gómez Lumbreras 2023 | US | 100,000 | -0.0343 | 149,497 | 342.2623 | -4,356,893 | -152,928 | 13,652,186,319 | 3 |
| Lee 2019 | US | 100,000 | -0.0444 | 49,748 | -29.7438 | -1,119,450 | -54,192 | 246,310,331 | 3 |
| **Semaglutide vs endoscopic sleeve gastroplasty** | | | | | | | | | |
| Haseeb 2024 | US | 100,000 | -0.0564 | 34,792 | -0.1546 | -617,318 | -40,428 | 5,814,835 | 3 |
| Saumoy 2023 | US | 100,000 | -0.0600 | 282,979 | NA* | -4,716,314 | -288,979 | 41,563,952 | 5 |
| **Semaglutide vs lifestyle intervention** | | | | | | | | | |
| Atlas 2022 | US | 100,000 | 0.900 | 220,138 | -1080.4513 | 244,598 | -130,138 | 3,582,101,545 | 4 |
| Kim 2022 | US | 100,000 | 0.1770 | 25,161 | 51.2021 | 142,075 | -7,451 | 135,582,091 | 4 |
| Lee 2019 | US | 100,000 | 0.0150 | 34,107 | 0.2568 | 2,278,795 | -32,611 | 180,324,765 | 3 |
| Lim 2023 | US | 100,000 | 0.1152 | 64,760 | 25.3015 | 562,198 | -53,241 | 56,901,460 | 4 |
| Saumoy 2023 | US | 100,000 | 0.5860 | 275,169 | NA* | 469,572 | -216,569 | 231,459,025 | 5 |
|  | | | | | | | | | |
| **Semaglutide vs liraglutide** | | | | | | | | | |
| Gómez Lumbreras 2023 | US | 100,000 | 0.3387 | 59,339 | 552.7022 | 175,215 | -25,473 | 15,660,028,669 | 3 |
| Kim 2022 | US | 100,000 | 0.1422 | 3,748 | 42.7192 | 26,362 | 10,470 | 163,072,906 | 4 |
| Lee 2019 | US | 100,000 | 0.0837 | -17,552 | 14.4532 | -209,583 | 25,926 | 385,885,488 | 3 |
| Mital 2023 | US | 100,000 | 0.0676 | -17,370 | 463.4527 | -256,767 | 24,134 | 4,429,055,296 | 3 |
| **Semaglutide vs naltrexone plus bupropion** | | | | | | | | | |
| Atlas 2022 | US | 100,000 | 0.6700 | 191,083 | 380.1393 | 285,198 | -124,083 | 2,553,713,820 | 4 |
| Gómez Lumbreras 2023 | US | 100,000 | 0.3986 | 198,573 | -371.5219 | 498,179 | -158,713 | 16,388,040,469 | 3 |
| Kim 2022 | US | 100,000 | 0.1630 | 22,562 | 100.4390 | 138,456 | -6,266 | 133,305,089 | 4 |
| **Semaglutide vs no treatment** | | | | | | | | | |
| Kim 2022 | US | 100,000 | 0.9215 | 28,696 | -342.6635 | 31,140 | 63,453 | 719,398,782 | 4 |
| Lee 2019 | US | 100,000 | 0.1334 | 37,087 | -23.8373 | 278,047 | -23,749 | 174,894,152 | 3 |
| Mital 2023 | US | 100,000 | 0.1617 | 92,189 | -256.2745 | 570,059 | -76,017 | 3,124,113,557 | 3 |
| **Semaglutide vs phentermine plus topiramate** | | | | | | | | | |
| Atlas 2022 | US | 100,000 | 0.4500 | 209500 | 1078.7261 | 481,384 | -171,623 | 1,691,807,272 | 4 |
| Gómez Lumbreras 2023 | US | 100,000 | 0.3011 | 187812 | 321.6775 | 696,654 | -179,633 | 17,263,313,949 | 3 |
| Kim 2022 | US | 100,000 | 0.1479 | 21290 | 103.6364 | 160,714 | -8,982 | 122,514,606 | 4 |
| Lee 2019 | US | 100,000 | 0.0444 | 26610 | 32.1363 | 709,598 | -27,058 | 173,390,794 | 3 |

*Covariance is not calculated in scenario 5 studies due to their varINB data being calculated using borrowed varINB values (see Appendix 3 Data preparation and currency conversion).

# Appendix 5: Comprehensiveness of performing sensitivity analyses among included studies

We examined the comprehensiveness of sensitivity analyses conducted in the included studies to ensure consistency and accuracy in the data collected. Parameters influencing the results of each study were identified. They are compared across studies to determine the list of key parameters through discussion within our research team. Each study was assessed to determine whether its sensitivity analysis incorporated these factors, and the range of values used for each factor were well justified. Studies that failed to either incorporate any of these factors or use unjustified range of values in their sensitivity analyses were classified as having performed incomplete sensitivity analyses.

| **Study** | **TX Cost** | **Efficacy** | **Treatment discontinuation** | **Weight regain following treatment discontinuation** | **Utility** | **Cost of complications** | **Comprehensiveness (%)** |
| --- | --- | --- | --- | --- | --- | --- | --- |
| Atlas, 2022 | Yes | Yes | Yes | Yes | Yes | Yes | 6/6 (100.0%) |
| Haseeb, 2024 | Yes | Yes | Yes | Yes | Yes | Yes | 6/6 (100.0%) |
| Kim, 2022 | Yes | Yes | Yes | Yes | Yes | Yes | 6/6 (100.0%) |
| Lim, 2023 | Yes | Yes | Yes | Yes | Yes | Yes | 6/6 (100.0%) |
| Mital, 2023 | Yes | Yes | Yes | Yes | Yes | Yes | 6/6 (100.0%) |
| Saumoy, 2023 | Yes | Yes | Yes | Yes | Yes | Yes | 6/6 (100.0%) |
| Gómez Lumbreras, 2023 | Yes | Yes | Yes | No | Yes | Yes | 5/6 (83.3%) |
| Lee, 2019 | Yes | Yes | Yes | Yes | No | No | 4/6 (66.7%) |
| Nuijten, 2021 | No | No | No | No | No | No | 0/6 (0.0%) |

# Appendix 6: Risk of bias of included studies

| **Author** | **PART A. Overall checklist for bias in economic evaluation** | | | | | | | | | | | **PART B. Model-specific aspects of bias in economic evaluation** | | | | | | | | | | | **Overall** |
| --- | --- | --- | --- | --- | --- | --- | --- | --- | --- | --- | --- | --- | --- | --- | --- | --- | --- | --- | --- | --- | --- | --- | --- |
|  |  |  |  |  |  |  |  |  |  |  |  | **I** | | | **II** | | | | | | | **III** |  |
|  | **Narrow perspective bias** | **Inefficient comparator bias** | **Cost measurement omission bias** | **Intermittent data collection bias** | **Invalid valuation bias** | **Ordinal ICER bias** | **Double-counting bias** | **Inappropriate discounting bias** | **Limited sensitivity analysis bias*** | **Sponsor bias** | **Reporting and dissemination bias** | **Structural assumptions bias** | **No treatment comparator bias** | **Wrong model bias*** | **Limited time horizon bias** | **Bias related to data identification** | **Bias related to baseline data** | **Bias related to treatment effects*** | **Bias related to quality-of-life weights (utilities)** | **Non-transparent data incorporation bias** | **Limited scope bias** | **Bias related to internal consistency** |  |
| Atlas, 2022 | Y | Y | Y | NA | Y | Y | Y | Y | Y | Y | NA | Y | Y | Y | Y | Y | Y | Y | Y | Y | Y | NA | **Low** |
| Gómez Lumbreras, 2023 | N | Y | Y | NA | Y | Y | Y | Y | P | Y | NA | Y | Y | Y | Y | Y | Y | Y | Y | Y | P | NA | **Moderate** |
| Haseeb, 2024 | N | P | Y | NA | Y | Y | Y | Y | Y | Y | NA | Y | Y | Y | P | Y | Y | Y | Y | Y | Y | NA | **Low** |
| Kim, 2022 | Y | Y | Y | NA | Y | Y | Y | Y | Y | Y | NA | P | Y | Y | Y | Y | Y | Y | Y | Y | Y | NA | **Low** |
| Lee, 2019 | N | Y | N | NA | Y | Y | Y | Y | P | Y | NA | Y | Y | Y | N | Y | Y | Y | P | Y | P | NA | **Moderate** |
| Lim, 2023 | Y | Y | Y | NA | Y | Y | Y | Y | Y | Y | NA | Y | Y | Y | P | Y | Y | Y | Y | Y | Y | NA | **Low** |
| Mital, 2023 | N | Y | N | NA | Y | Y | Y | Y | P | Y | NA | Y | Y | Y | Y | Y | Y | Y | Y | P | P | NA | **Moderate** |
| Nuijten, 2021 | Y | P | Y | NA | Y | Y | Y | Y | N | Y | NA | Y | N | Y | Y | Y | Y | Y | Y | Y | Y | NA | **High** |
| Saumoy, 2023 | N | P | Y | NA | Y | Y | Y | Y | Y | Y | NA | Y | Y | Y | Y | Y | Y | Y | Y | Y | Y | NA | **Low** |

**Abbreviations**: Y, Yes–low risk of bias; P, Partly Unclear–partly risk of bias; N, No-high risk of bias; NA, Not Available

The overall risk of bias was assessed based on three domains (blue highlighted columns), which were justified as most relevant to the overall validity assessment and the study context: 1) limited sensitivity analysis bias, 2) wrong model bias, and 3) bias related to treatment effects.

# Appendix 7: Pooled INB for each comparison

## 7.1 Semaglutide vs lifestyle intervention


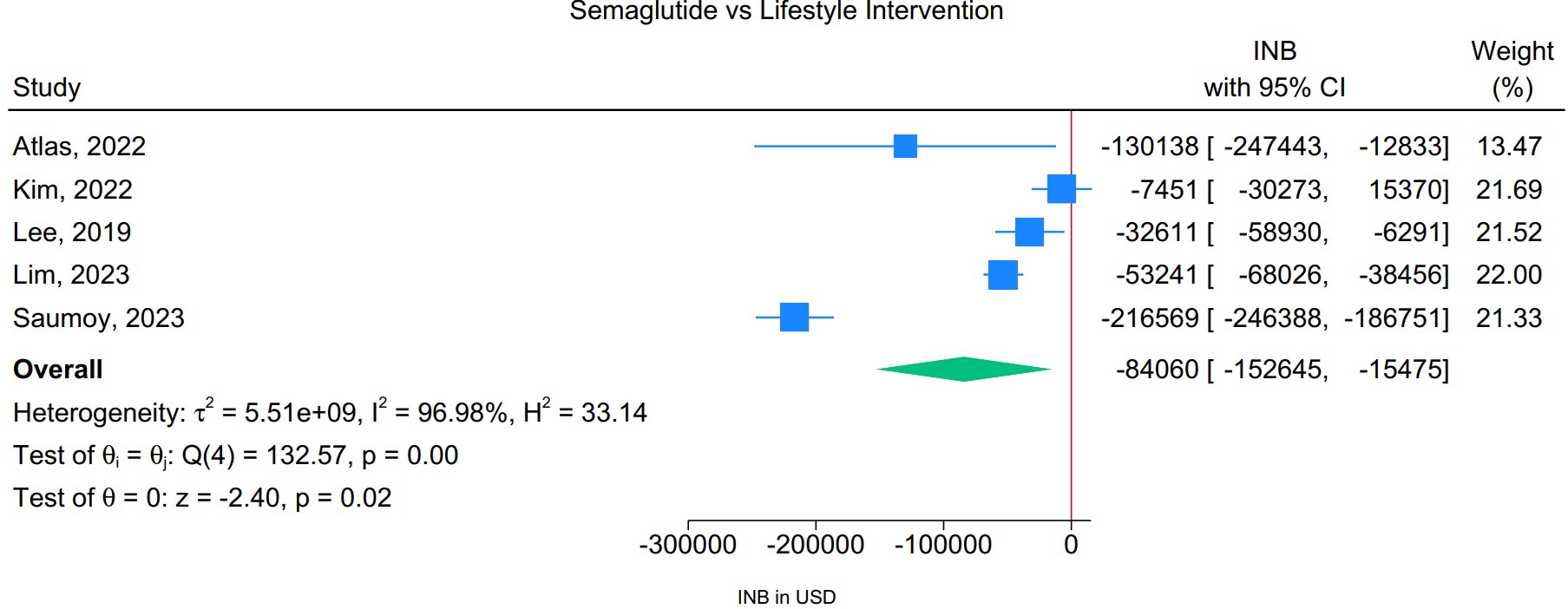


## 7.2 Semaglutide vs no treatment


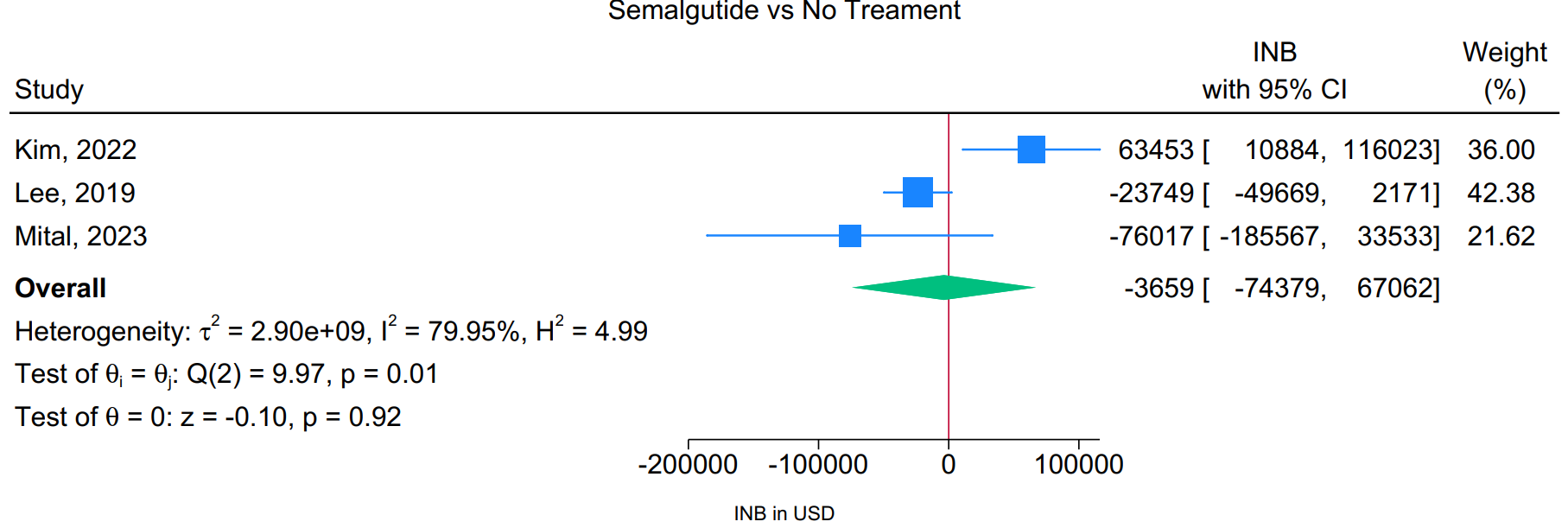


## 7.3 Semaglutide vs phentermine/topiramate


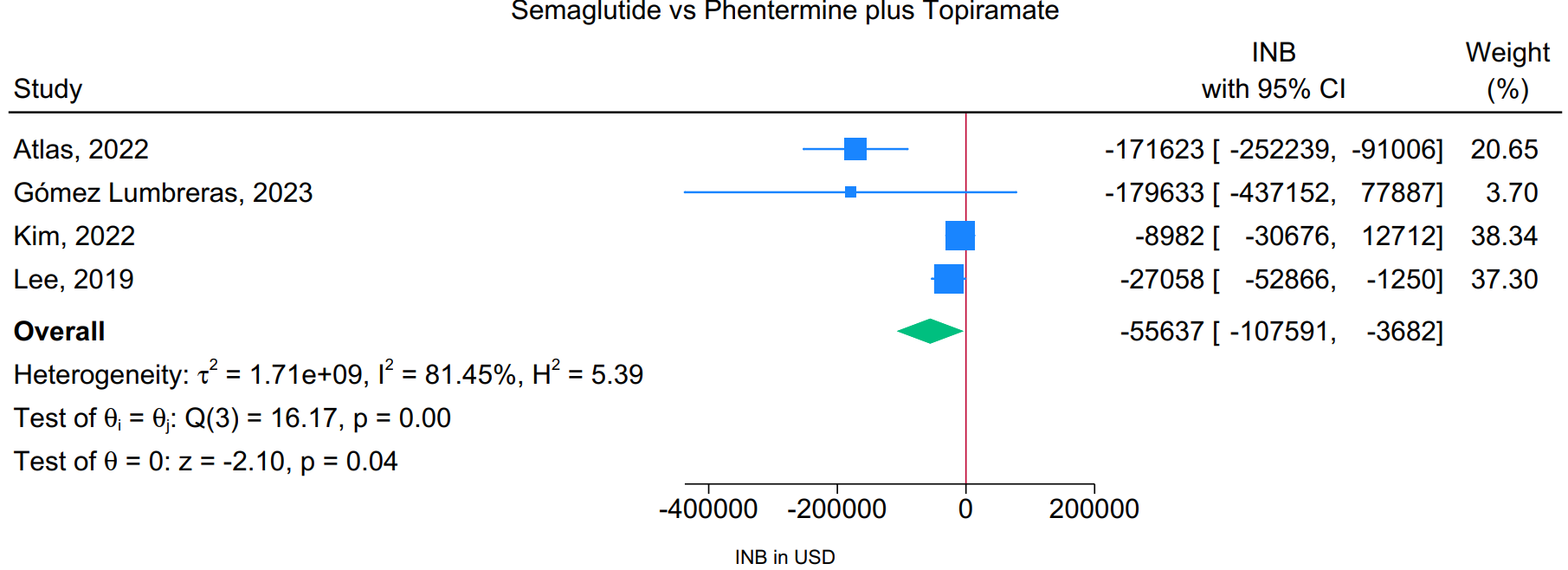


## 7.4 Semaglutide vs naltrexone/bupropion


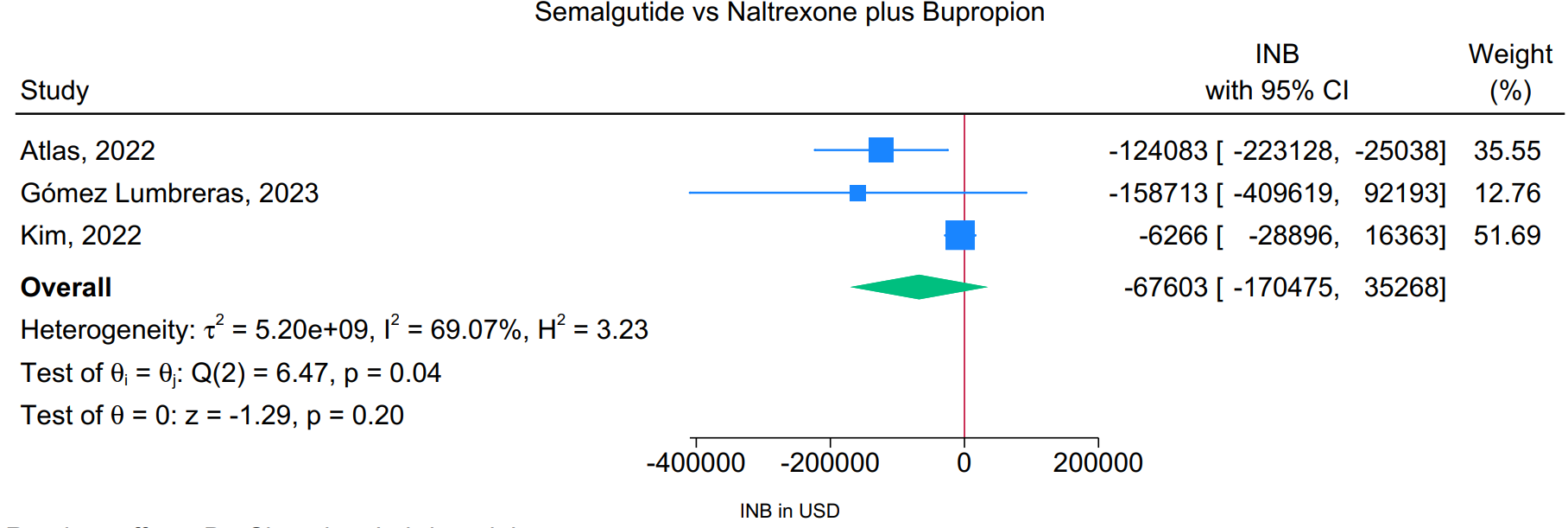


## 7.5 Semaglutide vs endoscopic sleeve gastroplasty


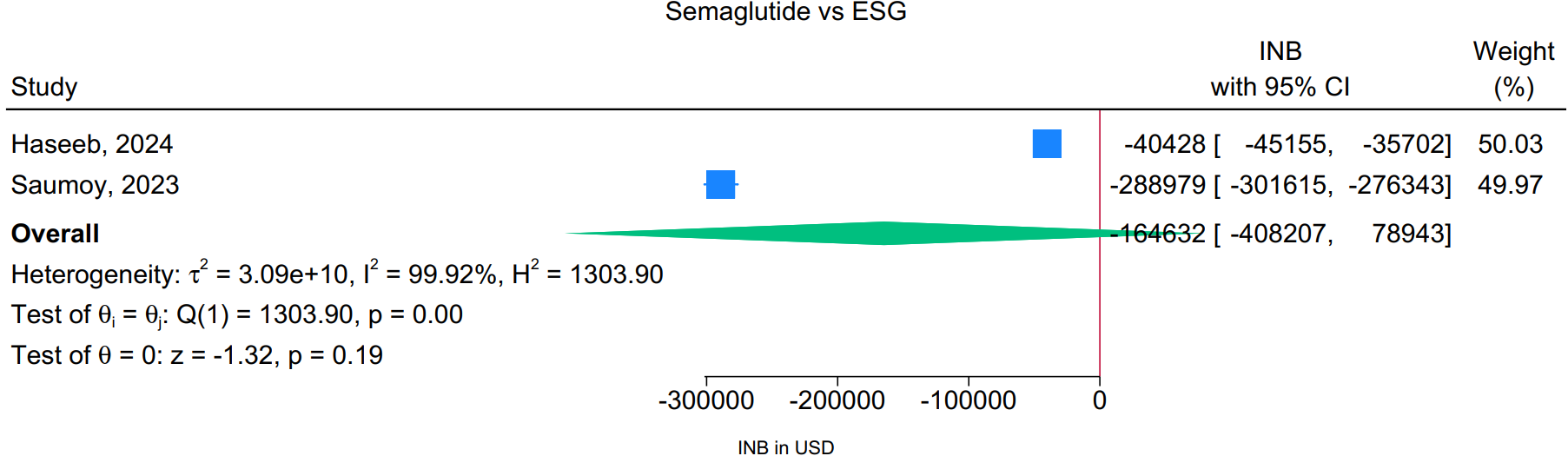


## 7.6 Semaglutide vs liraglutide


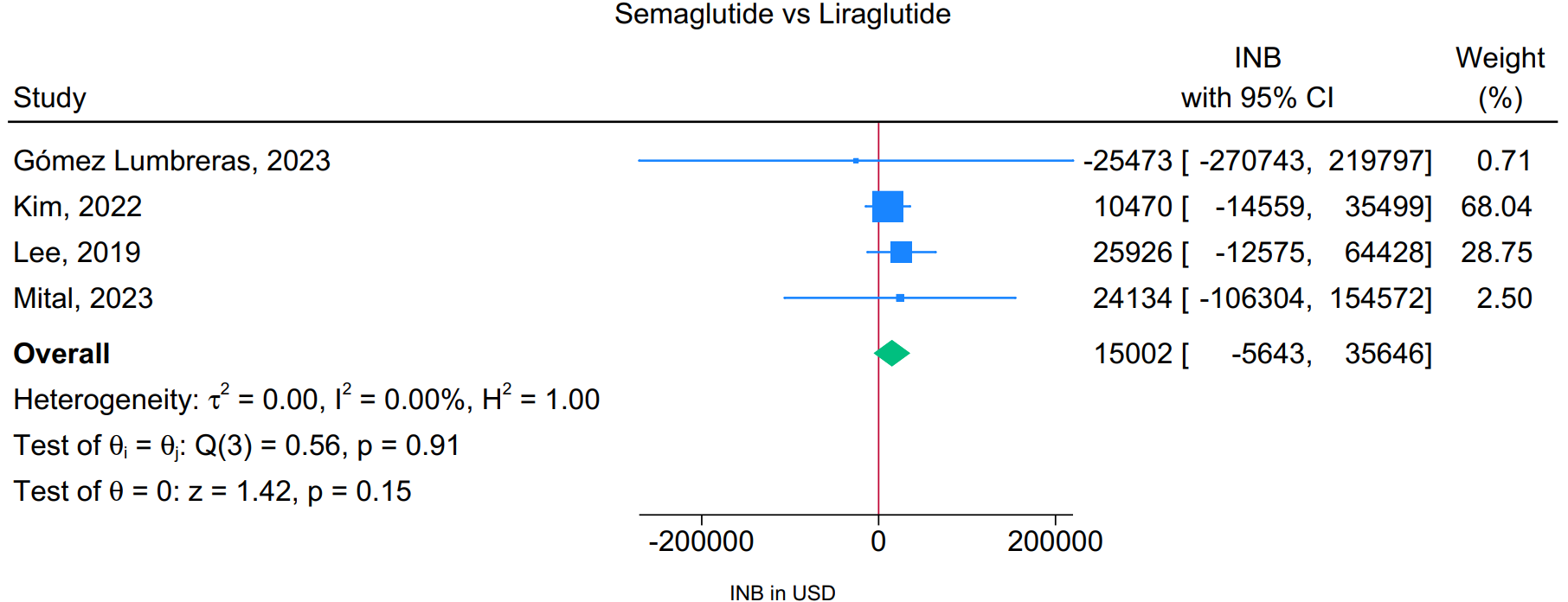


## 7.7 Liraglutide vs lifestyle intervention


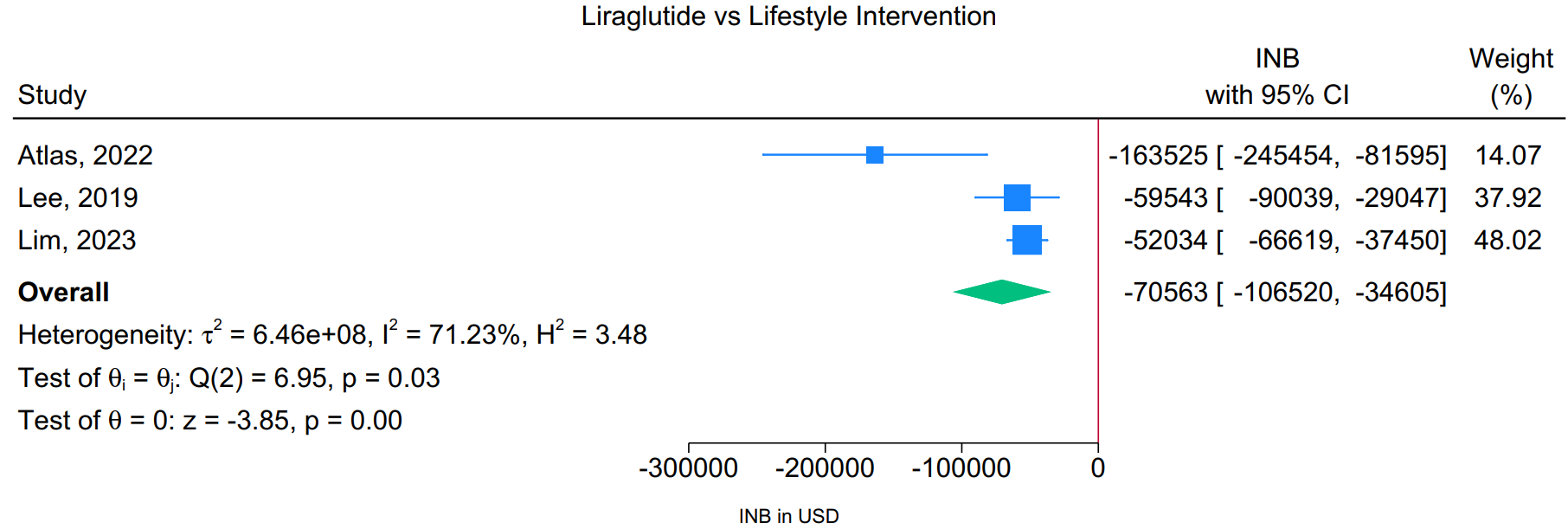


## 7.8 Liraglutide vs no treatment


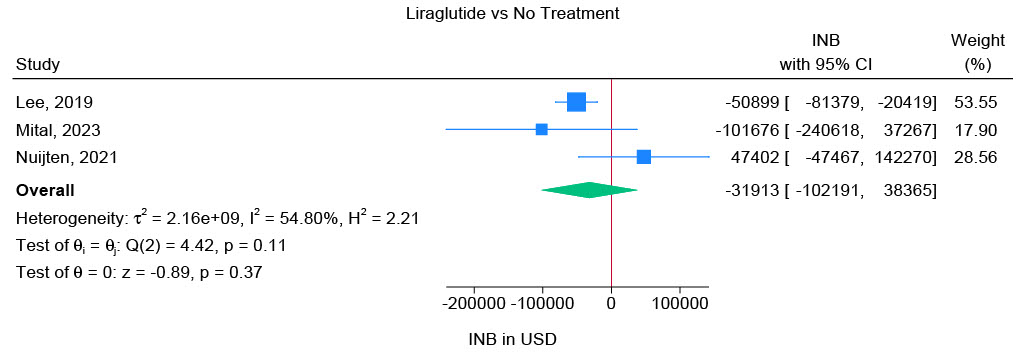


## 7.9 Liraglutide vs phentermine/topiramate


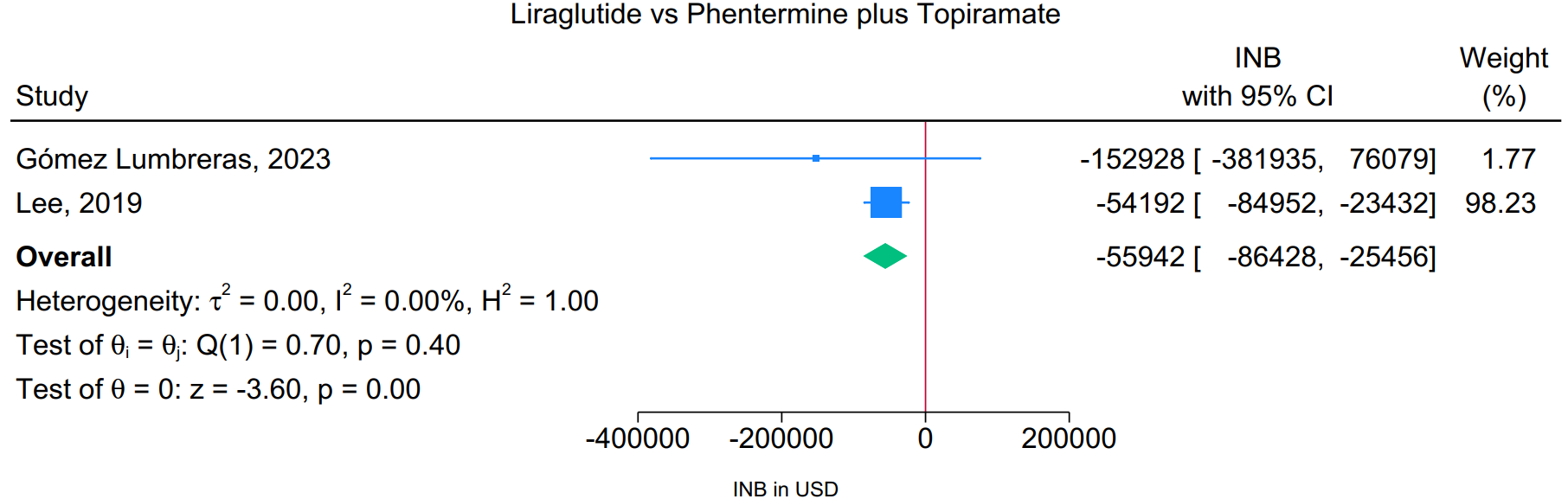


# Appendix 8: Sensitivity analyses

| **Comparisons** | **Main analysis** | | **Sensitivity analysis** | | | | | |
| --- | --- | --- | --- | --- | --- | --- | --- | --- |
|  | **All included studies** | **US-only** | **Variance from absolute values** | **Robust sensitivity analyses** | **Excluded Scenario 5** | **Excluded studies with less than 5 years treatment** | **Used Restricted Maximum Likelihood (REML) Model** | **WTP of $150,000 per QALY** |
| SEMA vs LI | -84,060  (-152,645 to -15,475;* I^2^ =96.98%, n=5) | NA | -85,969  (-177,337 to 5,398; I^2^ =98.81%, n=5) | -98,933  (-187,984 to -9,882;* I^2^ =97.64%, n=4) | -31,788  (-58298 to -5,278;* I^2^ =73.20%, n=4) | -105,740  (-191,894 to -19,585;* I^2^ =97.20%, n=4) | -84,779  (-162,797 to -6,761;* I^2^ =97.0%, n=5) | -67,857  (-131,373 to -4,341;* I^2^ =95.2%, n=5) |
| SEMA vs No Tx | -3,659  (-74,379 to 67,062; I^2^ =79.95%, n=3) | NA | NA | 2,339  (-133,291 to 137,969; I^2^ =80.24%, n=2) | Same results as the main analysis  (No S5) | -26,520  (-51,743 to -1,296;* I^2^ =0.00%, n=2) | -4,057  (-77,757 to 69,642; I^2^ =79.95%, n=3) | 16,873  (-81,585 to 115,331; I^2^ =81.3%, n=3) |
| SEMA vs PHEN+TOPI | -55,637  (-107,691 to -3,682;* I^2^ =81.45%, n=4) | NA | NA | -85,478  (-244,582 to 73,626; I^2^ =93.14%, n=2) | Same results as the main analysis  (No S5) | -107,105  (-232,066 to 17,856; I^2^ =83.80%, n=3) | -71,738  (-158,667 to 15,191; I^2^ =81.5%, n=4) | -42,568  (-93,738 to 8601; I^2^ =73.5%, n=4) |
| SEMA vs NAL+BUP | -67,603  (-170,475 to 35,268; I^2^ =69.07%, n=3) | NA | NA | -54,903  (-168,592, 58,786; I^2^ =80.64%, n=2) | Same results as the main analysis  (No S5) | -128,752  (-220,879 to -36,625; I^2^ =0.00%, n=2) | -66,698  (-167,166 to 33,769; I^2^ =69.1%, n=3) | -25,236  (-92,910 to 42,438; I^2^ =30.9%, n=3) |
| SEMA vs ESG | -164,632  (-408,207 to 78,943; I^2^ =99.92%, n=2) | NA | -164,700  (-408,275, 78,875; I^2^ =99.98%, n=2) | NA | -40,428  (-45,155 to -35,702;* I^2^ =NA, n=1) | Same results as the main analysis | -164,632  (-408,207 to 78,943; I^2^ =99.9%, n=2) | -167,536  (-411,289 to 76,217; I^2^ =99.9%, n=2) |
| SEMA vs LIRA | 15,002  (-5,643 to 35,646; I^2^ =0.00%, n=4) | NA | NA | 10,995  (-13,625 to 35,536; I^2^ =0.00%, n=2) | Same results as the main analysis  (No S5) | 24,647  (-11,868to 61,162; I^2^ =0.00%, n=3) | 15,002  (-5,643 to 35,646; I^2^ =0.00%, n=4) | 21,418  (-1,306 to 35,646; I^2^ =0.0%, n=4) |
| LIRA vs LI | -70,563  (-106,520 to -34,605;* I^2^ =71.23%, n=3) | NA | NA | -100,191  (-208,434 to 8,051; I^2^ =85.50%, n=2) | Same results as the main analysis  (No S5) | Same results as the main analysis | -79,296  (-134,849 to -23,743;* I^2^ =71.2%, n=3) | -64,305  (-94,171 to –34,438;* I^2^ =56.7%, n=3) |
| LIRA vs No Tx | -31,913  (-102,191 to 38,365; I^2^ =54.80%, n=3) | -53,230  (-83,002 to -23,458;* I^2^ =0.00%, n=2) | -47,166  (-91,136, -3,196;* I^2^ =11.72%, n=3) | NA | -31,788  (-58,298 to -5,278;* I^2^ =73.20%, n=1) | Same results as the main analysis | -31,906  (-102,268 to 38,456; I^2^ =54.8%, n=3) | -36,009  (-92,802 to 20,784; I^2^ =25.2%, n=3) |
| LIRA vs PHEN+TOPI | -55,942  (-86,428 to -25,456;* I^2^ =0.00%, n=2) | NA | NA | NA | Same results as the main analysis  (No S5) | Same results as the main analysis | -55,942  (-86,428 to -25,456;* I^2^ =0.0%, n=2) | -55,004  (-85,640 to -24,368;* I^2^ =0.0%, n=2) |

Data presented as incremental net benefit (95% confidence interval); *statistically significant

**Abbreviations:** BUP, bupropion; ESG, endoscopic sleeve gastroplasty; LI, lifestyle intervention; LIRA, liraglutide; NAL, naltrexone; NA, not available; PHEN, phentermine; QALY, quality-adjusted life year; SEMA, semaglutide; TOPI, topiramate; Tx, treatment; WTP, willingness to pay

# Appendix 9: Subgroup analyses

| **Comparisons** | **Subgroup analysis (all studies included – full dataset)** | | | | **Subgroup analysis (excluded studies with less than 5 years treatment)** | | | |
| --- | --- | --- | --- | --- | --- | --- | --- | --- |
|  | **Time horizon** | | **Subject age** | | **Time horizon** | | **Subject age** | |
|  | **<10 years** | **>10 years** | **Adolescent** | **Adults** | **<10 years** | **>10 years** | **Adolescent** | **Adults** |
| SEMA vs LI | -45,917  (-65,266 to -26,596;* I^2^ =44.26%, n=2) | -117,271  (-282,280 to 47,737; I^2^ =98.33%, n=3) | -53,241  (-68,026 to -38,456);* I^2^ =NA, n=1) | -94,379  (-201,133 to 12,375; I^2^ =97.70%, n=4) | Same results as the full dataset analysis | -192,737  (-268,440 to -117,034);* I² = 49.0%, n=2 | Same results as the full dataset analysis | -126,096  (-271,135 to 18,943); I² = 97.60%, n=3 |
| SEMA vs No Tx | -26,520  (-51,743 to -1,296;* I^2^ =NA, n=2) | 63,453  (10,884 to 116,023;* I^2^ = NA, n=1) | -76,017  (-185,567 to 33,533; I^2^ =NA, n=1) | 16,730  (-68,507 to 101,967; I^2^ =88.24%, n=2) | Same results as the full dataset analysis | NA | Same results as the full dataset analysis | -23,749  (-49,669 to 2,171); I² =NA, n=1 |
| SEMA vs PHEN+TOPI | -27,058  (-52,866 to -1,250;* I^2^ =NA, n=1) | -101,969  (-242,294 to 38,355; I^2^ =87.54%, n=3) | NA | Same results as the main analysis | Same results as the full dataset analysis | -172,338  (-249,272 to -95,403);* I² = 0.0%, n=2 | NA | -107,105  (-232,066 to 17,856); I² = 83.8, n=3 |
| SEMA vs NAL+BUP | NA | Same results as the main analysis | NA | Same results as the main analysis | NA | -128,752  (-220,879 to -36,625);* I² = 0.0%, n=2 | NA | -128,752  (-220,879 to -36,625);* I² = 0.0%, n=2 |
| SEMA vs ESG | -40,428  (-45,155 to -35,702;* I^2^ =NA, n=1) | -288,979  (-301,615 to -276,343);* I^2^ =NA, n=1) | NA | Same results as the main analysis | Same results as the full dataset analysis | Same results as the full dataset analysis | NA | Same results as the full dataset analysis |
| SEMA vs LIRA | 25,783  (-11,144 to 62,709; I^2^ =0.00%, n=2) | 10,100  (-14,800 to 34,999; I^2^ =0.00%, n=2) | 24,134  (-106,304 to 154,572; I^2^ =NA, n=1) | 14,767  (-6,141 to 35,675; I^2^ =0.00%, n=3) | Same results as the full dataset analysis | -25,473  (--270,743 to 219,797);  I² = 0.00%, n=1 | Same results as the full dataset analysis | 24,690  (-13,345 to 62,726);  I² = 0.00%, n=2 |
| LIRA vs LI | -53,432  (-66,859 to -40,275;* I^2^ =0.00%, n=2) | -163,525  (-245,454 to -81,959);* I^2^ =NA, n=1) | -52,034  (-66,619 to -37,450; I^2^ =NA, n=1) | -104,296  (-205,204 to -3,387; I^2^ =81.60%, n=2) | Same results as the full dataset analysis | Same results as the full dataset analysis | Same results as the full dataset analysis | Same results as the full dataset analysis |
| LIRA vs No Tx | -53,230  (-83,002 to -23,458;* I^2^ =0.00%, n=2) | 47,402  (-47,467 to 142,270; I^2^ =NA, n=1) | -101,676  (-240,618 to 37,267; I^2^ =NA, n=1) | -12,435  (-106,463 to 81,593; I^2^ =73.25%, n=2) | Same results as the full dataset analysis | Same results as the full dataset analysis | Same results as the full dataset analysis | Same results as the full dataset analysis |
| LIRA vs PHEN+TOPI | -54,192  (-84,952 to -234,32;* I^2^ =NA, n=1) | -152,928  (-381,935 to 76,079; I^2^ =NA, n=1) | NA | Same results as the main analysis | Same results as the full dataset analysis | Same results as the full dataset analysis | NA | Same results as the full dataset analysis |

Data presented as incremental net benefit (95% confidence interval); *statistically significant

**Abbreviations:** BUP, bupropion; ESG, endoscopic sleeve gastroplasty; LI, lifestyle intervention; LIRA, liraglutide; NAL, naltrexone; NA, not available; PHEN, phentermine; SEMA, semaglutide; TOPI, topiramate; Tx, Treatment

# Appendix 10: Publication bias assessment for each comparison

## 10.1 Semaglutide vs lifestyle intervention


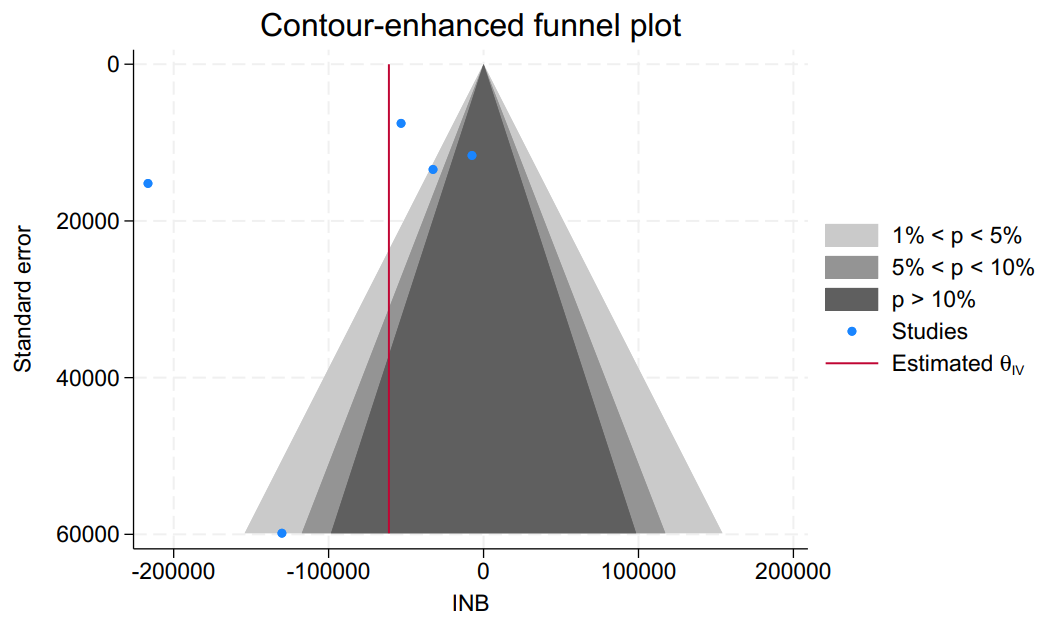


Egger’s test: p=0.605

## 10.2 Semaglutide vs no treatment


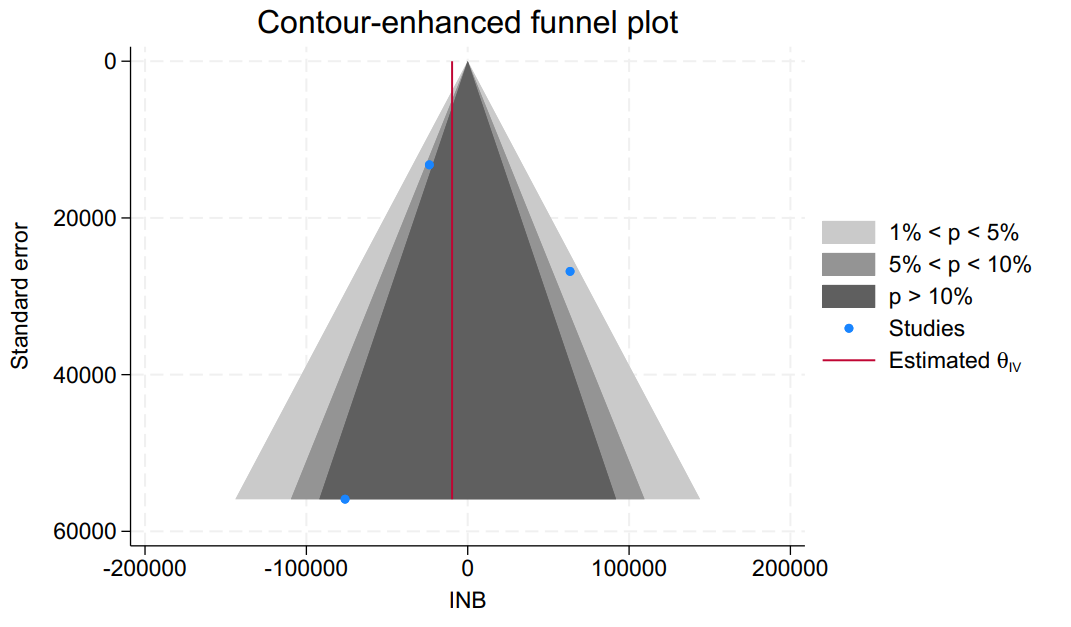


Egger’s test: p=0.884

## 10.3 Semaglutide vs phentermine/topiramate


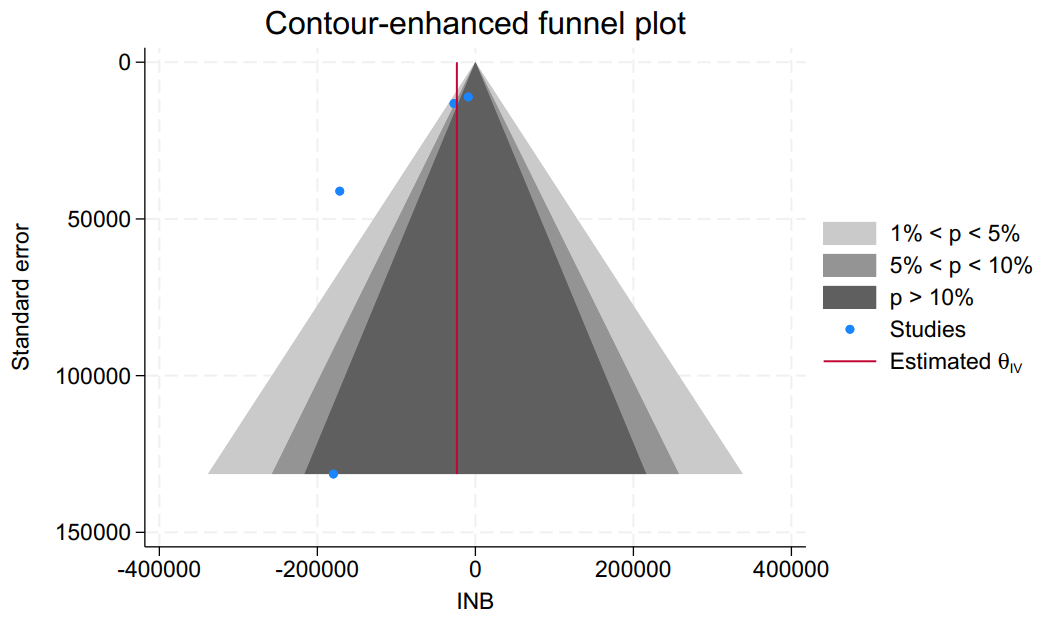


Egger’s test: p=0.180

## 10.4 Semaglutide vs naltrexone/bupropion


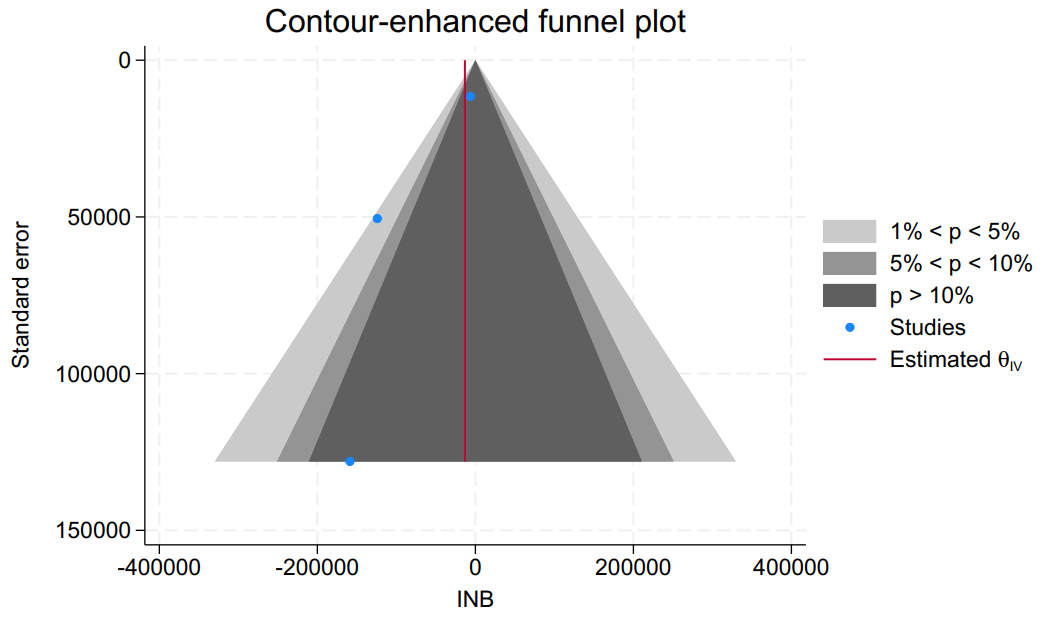


Egger’s test: p=0.258

## 10.5 Semaglutide vs endoscopic sleeve gastroplasty


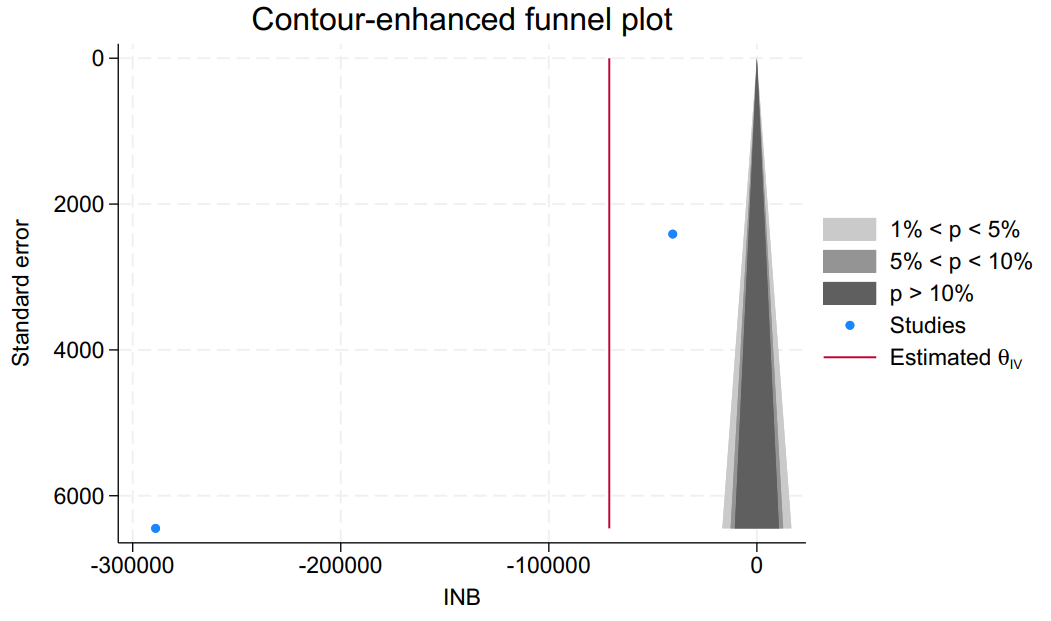


Egger’s test: p=Not available

## 10.6 Semaglutide vs liraglutide


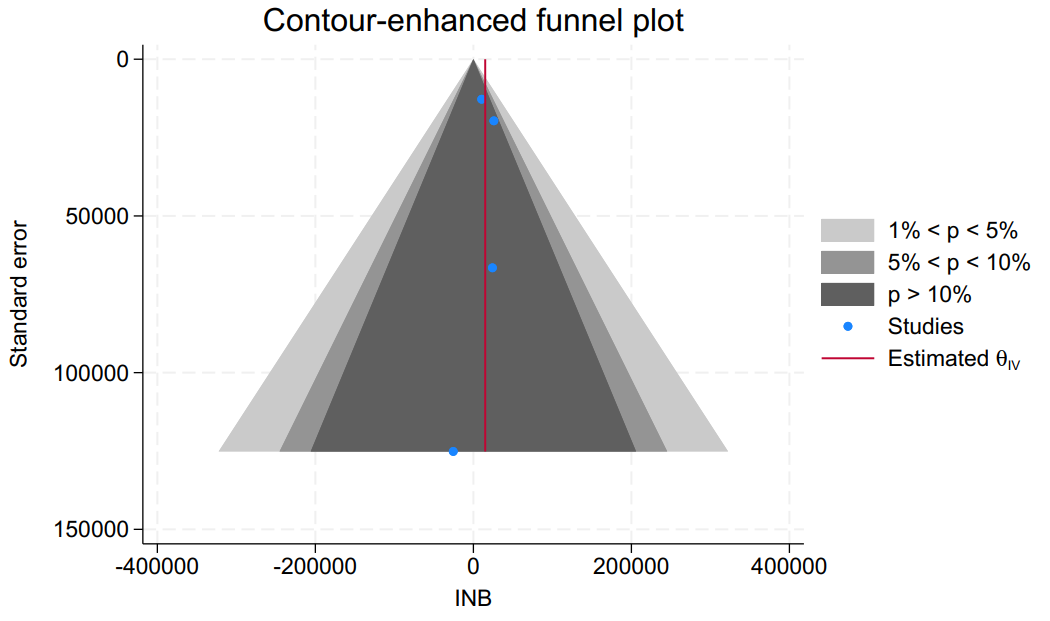


Egger’s test: p=0.983

## 10.7 Liraglutide vs lifestyle intervention


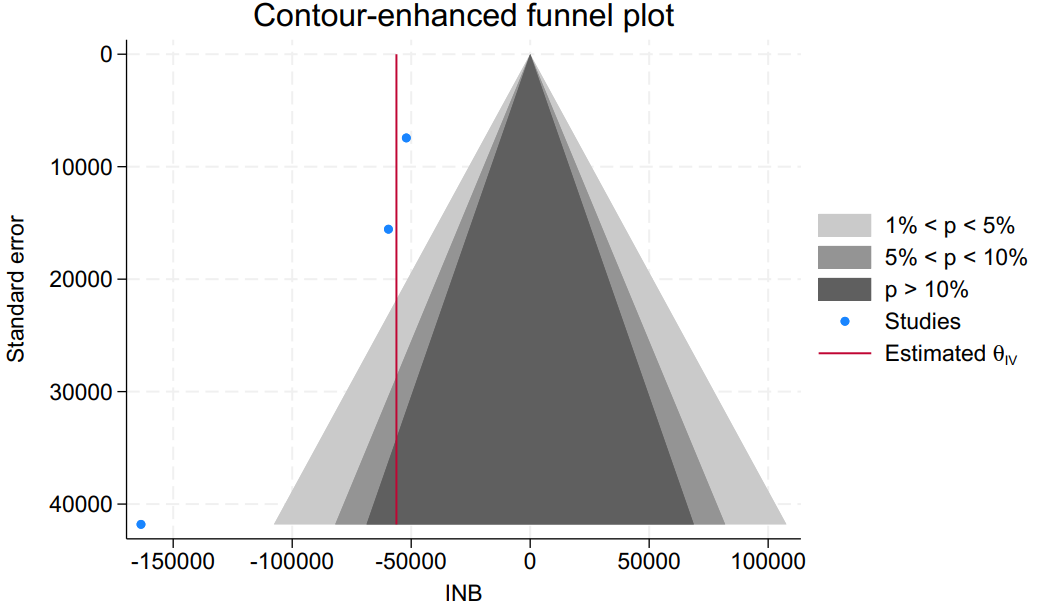


Egger’s test: p=0.242

## 10.8 Liraglutide vs no treatment


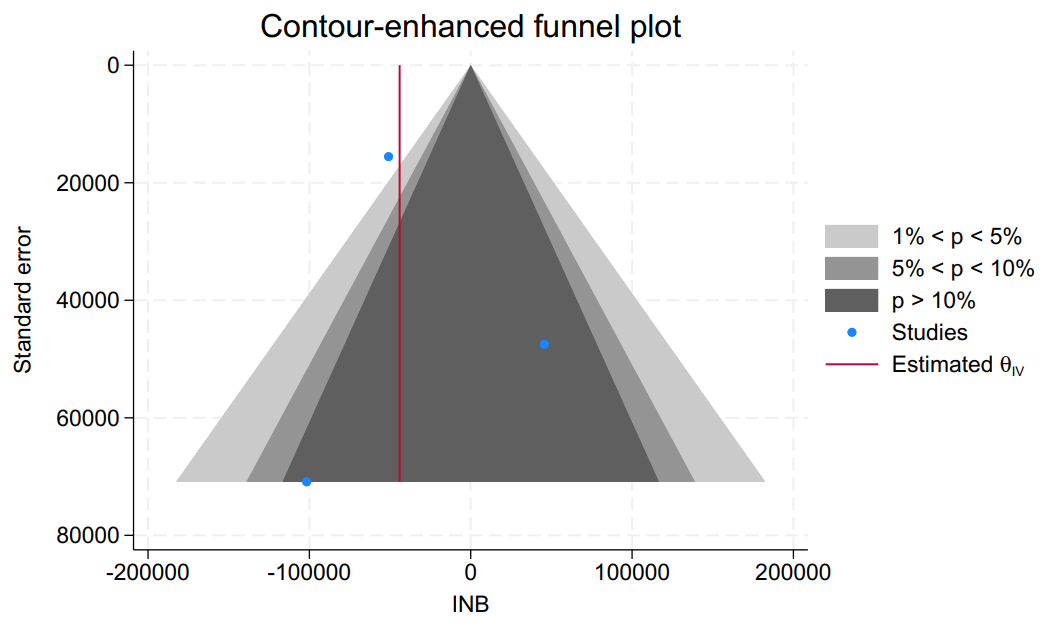


Egger’s test: p=0.793

## 10.9 Liraglutide vs phentermine/topiramate


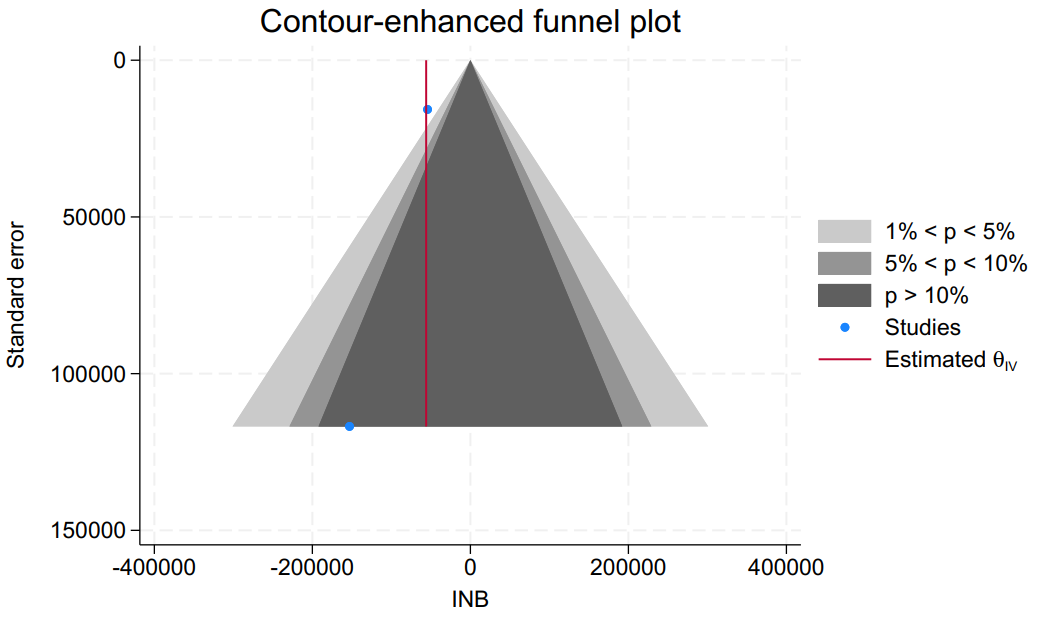


Egger’s test: p=Not available

1. Bagepally BS, Chaikledkaew U, Chaiyakunapruk N, Attia J, Thakkinstian A. Meta-analysis of economic evaluation studies: data harmonisation and methodological issues. BMC health services research. 2022 Feb 15;22(1):202. [↑](#footnote-ref-1)
